# Tertiary lymphoid structure drives allograft rejection via IFN-γ-JAK-STAT-dependent atypical memory B cell differentiation

**DOI:** 10.3389/fimmu.2025.1728290

**Published:** 2025-12-11

**Authors:** Lijie Ma, Shiming Gong, Jianchen Fang, Mingxuan Feng, Zhenzhen Zhan, Qiang Xia

**Affiliations:** 1 Department of Liver Surgery, Renji Hospital, School of Medicine, Shanghai Jiao Tong University, Shanghai, China; 2 Shanghai Institute of Transplantation, Shanghai, China; 3 Shanghai Engineering Research Centre of Transplantation and Immunology, Shanghai, China; 4 Department of Pathology, Renji Hospital, Shanghai Jiao Tong University School of Medicine, Shanghai, China

**Keywords:** tertiary lymphoid structure, living donor liver transplantation, post-transplant liver rejection, post-transplant liver fibrosis, atypical memory B cell

## Abstract

**Background:**

Tertiary lymphoid structure (TLS) is a neogenized, ectopic lymphoid aggregate found in infected, autoimmune and tumour tissues with an activated adaptive immune response. However, a comprehensive understanding of the pathological role, function, and formation of TLS in allograft rejection remains incomplete.

**Methods:**

We enrolled two large retrospective cohorts of liver biopsy (LB) after pediatric living donor liver transplantation (LDLT) and developed a deep learning pathomics (DLP) model. Gene expression profiles and corresponding clinical information of 590 cases were enrolled from three transcriptomic databases, including cohort-GSE193135 (n=337), cohort-GSE145780 (n=235), cohort-Renji (n=18). ESTIMATE, CIBERSORT, XCELL and MCP analyses were performed to visualize the immune landscape. Single-cell RNA-sequencing (scRNA-seq) analysis of 11 LBs after LDLT and multiplexed immunohistochemistry (mIHC) were performed to validate the discoveries of bioinformatics analysis.

**Results:**

We provided evidence that increased TLS in the liver was closely correlated with allograft rejection, fibrosis, and declined liver function. ScRNA-seq and *in vitro* co-culture analysis revealed that TLS form through the differentiation of atypical memory B (AtM B) cells via the JAK-STAT signalling pathway, stimulated by IFN-γ from exhausted CD8^+^ T effector memory (T_EM_) cells. The presence of TLS resulted in IgG accumulation, initiating pathological antibody-dependent cell-mediated phagocytosis (ADCP) of apoptotic hepatocytes by CD68^+^ macrophages. Preclinically, blocking JAK1/3 or knocking out *Lta* in mice limited TLS formation and attenuated allograft rejection in mouse orthotopic liver transplantation models, informing novel therapeutics for allograft rejection pathology.

**Conclusion:**

We proposed an efficient DLP model for predicting allograft rejection, and revealed an unexpected immunological mechanism of TLS in allograft rejection livers and clarified an IFN-γ-JAK-STAT-dependent circuit that could be targeted with drugs and transformed AtM B cells into potent instigators of hepatocellular injury in allograft rejection.

## Introduction

Post-transplant allograft rejection and fibrosis are two closely related complications of solid organ transplantation. Rejection occurs when the recipient’s immune system recognizes the donor’s allogeneic antigens and mounts an immune attack against the allograft, including hyperacute rejection, acute rejection (T-cell-mediated rejection (TCMR) and antibody-mediated rejection (AMR)), and chronic rejection (CR) (1). Fibrosis represents a scarring process involving fibroblast activation, collagen deposition, and parenchymal structural remodeling, arising from the combined effects of the aforementioned immune rejection and other non-immune factors, such as ischaemia-reperfusion injury, calcineurin inhibitor nephrotoxicity, viral infections, hypertension and metabolic disorders (2). Previous studies have revealed that, although T cell activation is sufficient for rejection pathogenesis (3), the transition to rejection critically depends on the involvement of other immune cells, especially B cells. However, the biological significance of B cells in allograft rejection remains a controversial issue, as diverse functionally B cell subsets represent dynamic changes in promoting both tolerance (4) and rejection (5). Intriguingly, it was observed that B cells and their downstream effector plasma cells (PCs) play a major role in acute and chronic AMR through alloantibody (6). However, the precise origins and roles of intrahepatic B cell subsets during the progression of allograft rejection need to be investigated.

Recently, increasing attention has been paid to the formation of tertiary lymphoid structures (TLS) in transplanted allografts as a possible cause of allograft dysfunction (7, 8). TLS are organized lymphoid aggregates that develop in non-lymphoid tissues in response to chronic inflammation or persistent antigenic stimulation (9). Importantly, the roles of TLS and B cells in these diseases are context-dependent and can be beneficial or detrimental. Notably, a strong association between TLS and maladaptive repair in aging models (10) and transplant recipients (11). Furthermore, TLS stages have been shown to correlate positively with the severity of kidney injury and inflammation, suggesting their potential as additional histological markers of tissue inflammation (12). Meanwhile, the presence of TLS in multiple liver cancers has also been documented, which was characterized as an *in situ* niche that supports antibody affinity maturation of antibody secreting cells (ASCs), in enhancing antigen presentation and the release of tumor-specific antibodies produced by ASCs, serving as prognosis indicators and positively correlate with favorable responses to immunotherapies (13–15). However, there is no evidence that TLS are formed in allografts after pediatric living donor liver transplantation (LDLT), especially immunoglobulins are derived from plasma cells (PCs) matured within allograft liver, specifically in the TLS niche. Meanwhile, a causal description of B cell-dominated TLS leading to progressive liver injury in allograft rejection to support this notion is still lacking. Furthermore, the mechanisms underlying B-cell differentiation and TLS formation during allograft rejection, as well as the strategies employed to restrain TLS-mediated hepatocyte injury, are not fully understood.

To comprehensively profile the characteristics of TLS and B cells, as well as their interactions within the allograft liver microenvironment, we investigated the immune landscape using single-cell RNA sequencing (scRNA-seq) and bulk RNA-seq, combined with an in-depth analysis and multiplex immunohistochemical (mIHC) staining in clinical liver biopsy (LB) cohorts and preclinical mouse liver transplantation (LT) models. Implementing these strategies enabled us to identify a close association between the presence of TLS and declined liver function. We also investigated the molecular processes underlying TLS formation in allograft rejection and the impact of their presence on the enhanced adaptive response and hepatocyte apoptosis, which provided potential pharmacological targets for preventing allograft rejection.

## Materials and methods

### Patient cohort and study design

Patients and cohorts stratification were summarized as follows: (1). For DLP model construction, we enrolled a retrospective, single-center study involving 847 pediatrics (Discovery LDLT WSI cohort) who underwent LDLT and received their first LB between October 2006 and December 2024 at the Department of Liver Surgery at Renji Hospital, Shanghai Jiao Tong University School of Medicine; (2). We further enrolled an internal validation LDLT WSI cohort (n=117) who underwent LDLT and received their first LB between October 2006 and December 2024 for further validation; (3). We also established a bulk RNA-seq cohort (n=18) and scRNA-seq cohort (n=11) through LB after LDLT between January 2023 and December 2024, which receiving three types of Tacrolimus doses at the time of LB (pediatric receptor receiving heterogeneous Tacrolimus doses after LDLT based on distinct Tacrolimus metabolism gene, liver function and allograft status). A high Tacrolimus dose was indicated as more than 1.25 mg/day (or 0.06 mg/kg); a low dose was indicated as less than 0.2 mg/day (or 0.01 mg/kg); tolerance was indicated as an off-medication status, which was always classified as operational immune tolerance due to an unexpected infection; rejection was indicated as definitive pathological diagnosis of rejection with LB; (4). We also collected another 15 LBs (including 5 post transplantation liver fibrosis (PTLF), 5 post transplantation liver rejection (PTLR) and 5 normal allograft LBs) between January 2023 and December 2024.

The research described in this manuscript involving clinical data collection and analysis is approved by the Ethical Committee’s Institutional Review Board of Renji Hospital (KY2022-117-B), Shanghai Jiao Tong University School of Medicine, in compliance with the Declaration of Helsinki. The inclusion criteria was described as followed: pediatrics (under 18 years of age) were eligible to participate in the study if they underwent the first allograft LB scheduled after LDLT at our facility. The exclusion criteria was described as followed: pediatrics with a history of multiple organ transplants, re-transplantation, or a malignant tumor diagnosis were excluded. All recipients received a calcineurin inhibitor (CNI) based immunosuppressive (IS) strategy involving Tacrolimus or cyclosporin after LDLT. Data collection encompassed the following: clinical information; pathology information; the reason for the LB; the time of LB after LDLT; the dose of the immunosuppressive (IS) inhibitor at the time of LB; the pathological report of the LB; and the liver function index (including ALT, AST, and TB) (**Supplementary Figure S1A**, **Supplementary Tables S1**, **2**).

### Histopathology evaluation

Allograft biopsy samples were centrally assigned to the following categories based on the 2022 update of the Banff criteria (16). To facilitate subsequent pathological analysis, we categorized the histopathological status of liver biopsy specimens into three groups: Normal (normal biopsy or non-specific changes); Rejection (including T-cell mediated rejection (TCMR), also defined as acute cellular rejection (ACR), borderline and antibody mediated rejection (AMR)); and Undefined (where the rejection reaction could not be ruled out). An experienced transplant pathologist assessed these categories by examining the Banff lesions and final diagnoses within the pathology reports. Rejection activity index (RAI) 1–2 points indicated Normal or no ACR; 3 points indicated borderline/uncertain ACR; 4–5 points indicated mild ACR; 6–7 points indicated moderate ACR; 8–9 points indicated severe ACR (**Supplementary Table S3**).

### Pathological examination of TLS

TLSs were evaluated on the whole H&E-stained slides and classified according to their maturation status as previously described (17). Two pathologists who were blinded to the pathological diagnoses and allograft outcomes evaluated all available scanned whole-slide images of haematoxylin and eosin (H&E)-stained LB slides from our centre that were used for routine pathological diagnosis. TLSs were evaluated on the whole H&E-stained slides and classified according to their maturation status as followed: (i) Mature TLS: secondary lymphoid follicles with germinal centre (GC) formation. GCs are highly dynamic structures consisting of a network of follicular dendritic cells (FDCs) filled with centroblasts and centrocytes. The GC can be divided into two zones: the dark zone (DZ), which is dominated by centroblasts, and the light zone (LZ), which contains centrocytes and FDCs. Extensive apoptosis and phagocytosis phenomena could be observed in both DZ and LZ compartments; and (ii) immature TLS: loose, ill-defined clusters of lymphoid aggregates or oval-shaped clusters of lymphocytes without a GC. The allograft LB was stratified according to the existence and maturation status of TLS on each patient’s H&E-stained slides: TLS-mature allografts contain at least one mature TLS; TLS-immature allografts contain at least one immature TLS but no mature TLS; and TLS-negative allografts contain neither mature nor immature TLS.

### Multiplex immunohistochemistry staining

Multiplex immunohistochemistry (mIHC) was performed according to the manufacturer’s instructions (Opal^®^ Reagent Kit, Akoya Biosciences), as previously described (18). This involved a sequential application of antibodies and fluorescent dyes to the following panels (**Supplementary Table S4**). The stained slides were scanned at low magnification (10x) under fluorescent conditions using the Vectra Polaris 1.0.13 imaging system (Akoya Biosciences), and then examined at high magnification (20x) for each core. Digital image analysis software (InForm 2.4.0, Akoya Biosciences) was then used to analyse each sample. Marker colocalisation was used to identify specific cell phenotypes from each mIHC group. The density of each cell phenotype was quantified and the final data are presented as cell numbers/mm². Spatial cell distribution analysis was conducted using the spatial analysis module of HALO software (Indica Labs, Albuquerque, NM, USA). Each TLS was divided into two maturation: Mature TLS: primary follicular lymphoid (PFL) TLS: CD20+CD21+CD23-; second follicular lymphoid (SFL) TLS: CD20+CD21+CD23+; (ii) Immature TLS: aggregated (Agg) TLS: CD20+CD21-CD23-.

### Multiple instance learning -based deep learning pathomics model

These patch-level likelihoods were then aggregated in an ensemble classifier to obtain the WSI-level prediction (19). The raw image data were preprocessed using the Otsu method to remove meaningless background. Our MIL-based DLP model produced two predictions: one at the level of individual patches and one at the level of the whole slide image (WSI). Due to the heterogeneity of the liver microenvironment, the WSI was first divided into small, non-overlapping patches (512x512 pixels), which are referred to as labeled tiles. For DLP model construction, 847 WSIs and 152285 patches were collected from the Discovery LDLT WSI cohort (n=847 pediatrics). To make the patch-level prediction, we trained residual convolutional neural networks (ResNet-18 and Inception_V3) to calculate the likelihood of each patch within a MIL framework. In this paradigm, the patches were assigned the label of the WSI. We optimized the network using the mini-batch gradient descent method with binary cross-entropy (BCE) loss. We developed two independent MIL methods to aggregate the patch likelihoods: The Patch Likelihood Histogram (PALHI) pipeline and the Bag of Words (BoW) pipeline. The former was inspired by the histogram-based method, while the latter was inspired by the vocabulary-based method. In the PALHI pipeline, a histogram of patch likelihood occurrence was used to represent the WSI. In the BoW pipeline, each patch was mapped to a TF-IDF floating-point variable, and a TF-IDF feature vector was computed to represent the WSI. Traditional machine learning classifiers were then trained using these feature vectors to predict MS status for each WSI. In the PALHI pipeline, Extreme Gradient Boosting (XGBoost), a type of gradient-boosted decision tree, was employed. Naive Bayes was used in the BoW pipeline. During training of the WSI-level classifier, hyperparameters were determined using cross-validation on the training set, with WSI-level AUROC serving as the performance metric. The PALHI and BoW classifier results were then combined at the WSI level to obtain the final prediction.

### Identification of pathomics signatures

We developed a nuanced pathomics signature by integrating patch-level predictions, probability histograms and TF-IDF features in order to create individualised patient profiles. To refine the selection of features, we employed the LASSO method and the Pearson correlation coefficient, retaining only the most highly correlated features from each pair (those with a correlation exceeding 0.9). The model incorporates various deep learning methodologies, such as Support Vector Machine (SVM), Multi-Layer Perceptron (MLP), K-Nearest Neighbour (KNN), LightGBM, Gradient Boosting and AdaBoost. Together, these techniques form the pathomics signature.

### Visualization of rejection-related features through gradient-weighted class activation mapping

To unveil the”black box” of the diagnostic model, we first applied the Grad-CAM (20) to visualize the rejection-related features. In this section, we demonstrate how Inter-MIL improves interpretability by providing insight into the optimization of features at various scales, from fine-grained, tile-level features to global, biologically relevant features. We use different models to generate gradient activation maps for individual tiles using Grad-CAM.

### Acquisition of public RNA-seq datasets

Microarray data with clinical information were obtained from GSE193135 (PTLF, n=337, ClinicalTrials.gov, #NCT03193151) and GSE145780 (PTLR, n=235) for elucidating cross-omics correlations and potential molecular mechanisms.

### Calculation of differentially expressed genes and functional enrichment analysis

Gene Ontology (GO) and Kyoto Encyclopedia of Genes and Genomes (KEGG) enrichment analyses were performed using the ClusterProfiler R package to identify enriched functional pathways in the two subgroups. The R package limma was used to calculate the genes that were differentially expressed under different conditions. Genes with an adjusted p-value of less than 0.05 and a fold change greater than 1.5 (log_2_ fold change (log_2_FC) greater than 0.58) were considered to have significantly increased expression, whereas those with an adjusted p-value of less than 0.05 and a fold change less than 0.67 (log_2_FC less than -0.58) were considered to have significantly decreased expression. Another method of functional enrichment analysis, gene set variation analysis (GSVA), was employed using the ClusterProfiler R package to analyse differentially regulated pathways. The gene sets for GSVA analysis were downloaded from the Molecular Signatures Database (MSigDB, v7.1) at the Broad Institute.

### Mouse orthotopic liver transplantation model

Eight- to ten-week-old male C57BL/6J and C3H/He mice weighing 23 ± 2 g were used as donors and recipients, respectively. All animals were procured from Beijing Vital River Laboratory Animal Technology Co., Ltd. and were housed in a specific pathogen-free environment. Animal experiments were approved by the Shanghai Jiao Tong University School of Medicine Animal Care & Use Committee, which are designed and reported following the ARRIVE guidelines. MOLT was performed on three groups of mice: (i) the control group (C57 (donor) to C3H (receptor)), (ii) the LTA group (Lta^-/-^ (donor) to C3H (receptor)), and (iii) the inhibitor group (C57-C3H combined with a JAK1/3 inhibitor (Tofacitinib, HY-40354, MCE), used in the next day after LT, 10 μg/g/day). Each group contained five mice. Liver samples were collected 14 days after surgery. Under inhalation anaesthesia, the donors were injected with heparin and cross-incised to expose the liver. The detached liver was perfused and immersed in a cold University of Wisconsin (UW) solution before being orthotopically implanted into the abdomen of the recipient mouse. The suprahepatic inferior vena cava was connected using a continuous suturing technique. The portal vein and infrahumanic inferior vena cava were reconstructed using the ‘double cuff’ technique. The common bile duct was associated with an indwelling biliary stent. The hepatic artery was not reconstructed. Following transplantation, standard rodent chow and sterilized water were made available ad libitum.

### scRNA-seq data processing

Raw reads were processed to generate gene expression profiles using CeleScope v1.5.2 (Singleron Biotechnologies) with default parameters. Briefly, Barcodes and UMIs were extracted from R1 reads and corrected. Adapter sequences and poly A tails were trimmed from R2 reads and the trimmed R2 reads were aligned against the GRCh38 (hg38) {GRCm38 (mm10)} transcriptome using STAR (v2.6.1b). Uniquely mapped reads were then assigned to exons with FeatureCounts (v2.0.1). Successfully Assigned Reads with the same cell barcode, UMI and gene were grouped together to generate the gene expression matrix for further analysis.

### Dimensionality reduction, clustering, and cell type annotation

Scanpy v1.8.1 was used for quality control, dimensionality reduction and clustering under Python 3.7. For each sample dataset, the expression matrix was filtered using the following criteria: 1) cells with a gene count of less than 200 or in the top 2% of gene counts; 2) cells with a UMI count in the top 2%; 3) cells with a mitochondrial content of 20%; and 4) genes expressed in fewer than five cells. After filtering, cells were retained for downstream analysis, with an average of 1,398 genes and 3,697 UMIs per cell. The raw count matrix was normalized by total counts per cell and transformed logarithmically into a normalized data matrix. The top 2,000 variable genes were selected by setting flavour = ‘seurat’. Principal component analysis (PCA) was performed on the scaled variable gene matrix and the top 20 principal components were used for clustering and dimensional reduction. The Louvain algorithm was used to separate the cells into 12 clusters, setting the resolution parameter at 0.8. The cell clusters were visualized using Uniform Manifold Approximation and Projection (UMAP) and t-Distributed Stochastic Neighbour Embedding (t-SNE).

### Cell-cell interaction analysis: CellPhoneDB

Cell-to-cell interactions (CCIs) between activated B cells (AtM B) and T cell types were predicted based on known ligand-receptor pairs using CellPhoneDB (v2.1.0) (21). The permutation number used to calculate the null distribution of average ligand-receptor pair expression in randomised cell identities was set to 1000. The expression of individual ligands or receptors was thresholded using a cutoff based on the average log gene expression distribution for all genes across each cell type. Predicted interaction pairs with a p-value of less than 0.05 and an average log expression greater than 0.1 were considered significant and visualised using heatmap_plot and dot_plot in CellphoneDB.

### Transcription factor regulatory network analysis (SCENIC)

The transcription factor network was constructed using the SCENIC R toolkit (22) and the scRNA expression matrix, as well as the transcription factors from AnimalTFDB. The GENIE3 package was used to predict a regulatory network based on the co-expression of regulators and targets. The RcisTarget package searched for transcription factor binding motifs in the given data.

### B and T cell subsets isolation and co-culture experiment

Healthy blood B cells were isolated from PBMC using anti-CD19 microbeads (Miltenyi Biotec, Cat #130050301) in a MACS column purification system (Miltenyi Biotec). Using FACS Arial II (BD Bioscience), T cell subset was sorted as CXCR6^+^CD8^+^T cells. The purity of enriched and sorted cells was routinely verified to be more than 95%. The collected cells were either immediately used for functional co-culture experiments. For the CXCR6^+^CD8^+^ T_EM_ cells and B cell co-culture experiment, sorted CXCR6^+^CD8^+^T cells were stimulated with CD3/CD28 beads and were further plated with isolated total B cells from healthy blood at a ratio of 1:5 in the final volume of 200 µl for 7 days. Phenotypic staining of AtM B cells was performed.

### Flow cytometry analysis

Peripheral blood mononuclear cells (PBMCs) were stained with 7-AAD in order to filter out dead cells. For surface phenotype staining, the cells were incubated with antibodies in MACS buffer at room temperature for 15 minutes. The data were acquired using a FACSCelesta flow cytometer (BD Biosciences) and analysed using FlowJo software (version 10.8.1, BD Biosciences).

### Western blotting

The protein lysates from cells were prepared with Protein Extraction Reagent buffer (Thermo Fisher Scientific) containing PMSF (1 mM). After complete cell lysis, collect the cell lysate into pre-chilled centrifuge tubes using a cell scraper. All of these sample-handling steps should be performed on crushed ice. Following this, centrifuge the samples at 12000 rpm for 10 mins at 4 °C, and use the resulting supernatant for subsequent experiments. After the standard procedures for western blotting, the proteins were transferred to polyvinylidene difluoride membranes. Then, the membranes were incubated with primary antibodies against anti-GAPDH (1:1000 dilution), anti-Jak1 (1:1000 dilution), anti-p-Jak1-Tyr1034/1035 (1:1000 dilution), anti-Jak3 (1:1000 dilution), and anti-p-Jak3-Tyr980/981 (1:1000 dilution) overnight, followed by incubation with anti-rabbit IgG HRP-linked (1:2000 dilution) or anti-mouse IgG HRP-linked (1:2000 dilution) secondary antibodies for visualization with an imaging system.

### Statistical analysis

All statistical analyses were performed using R, version 4.3.1. Categorical data were evaluated using either the chi-squared test or Fisher’s exact test. Differences in quantitative data between the two groups were analyzed using a Student’s t-test. The quantitative results are presented as means ± SD or SEM. The Kaplan-Meier method was used to estimate allograft rejection, with survival curves plotted. Statistical significance was set at p < 0.05.

## Results

### Develop an interpretable DLP model to predict allograft rejection

To explore the key pathological alterations across allograft rejection development (Normal-Undefined-Rejection, **Supplementary Tables S1**-**3**), we first enrolled a retrospective cohort study of LB after pediatric LDLT (n=847, training to testing ratio: 7:3; **Supplementary Figure S1A**, **Supplementary Tables S1**, **2**) and aimed to develop a deep learning pathomics (DLP) model. The DLP model consisted two consecutive stages: patch-level prediction and WSI-level prediction. All WSIs were first cropped into multiple small ‘tiles’ at a magnification of 40×. These were then color standardized and subsequently fed into a residual convolutional neural network (ResNet-18) to obtain the patch-level prediction (**Supplementary Figure S1B**). The visualization of the heat map and GRAD-CAM provided the patch-level prediction for the normal, undefined and rejection groups, respectively (**Supplementary Figure S1C**). Two independent MIL pipelines were trained to integrate the multiple patch-level predictions into a diagnosis score at the WSI level: the PALHI pipeline and the BoW pipeline. Ensemble learning was employed to predict the immune status of allografts and obtain the optimal convex combination of the two MIL methods (**Supplementary Figure S1D**).

**Figure 1:**
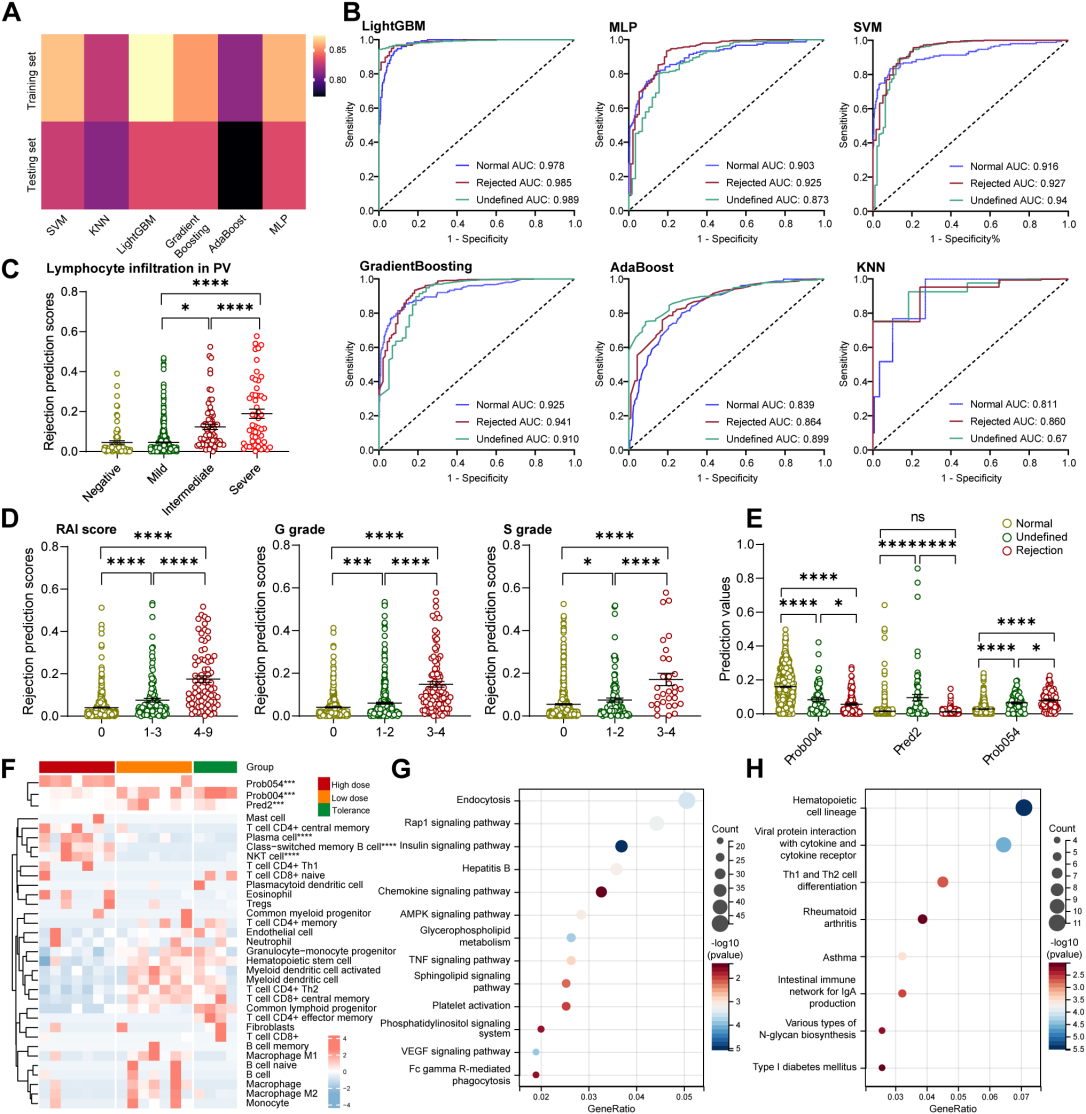
Develop an interpretable DLP model to predict allograft rejection. **(A)** Heatmap showed different accuracy among six deep learning methods in the discovery cohort (including training and validation sets). **(B)** Receiver operating characteristic curves of six deep learning methods for assessing allograft immune status: normal (purple line), rejected (red line), and undefined (green line) in the training set. **(C)** The dot plot showing the rejection prediction score showed a progressive increase from negative to severe, based on lymphocyte infiltration in the portal vein area. **(D)** The dot plot showing the rejection prediction score shows a progressive increase from 0 to 4–9 based on the RAI score, G grade, and S grade. **(E)** Dot plot showing the prediction values of Prob054, Prob004, and Pred2 among the three allograft status. **(F)** Heat map with clustering analysis showing the correlation between the immune landscape and top pathological signatures in three IS groups (high, low, and tolerance). Each column corresponds to the liver biopsy sample. **(G)** Bubble diagram depicting the signaling pathways enriched by KEGG analysis according to up-regulated DEGs in the high-dose group. **(H)** Bubble diagram depicting the signaling pathways enriched by KEGG analysis according to up-regulated DEGs in the low-dose and tolerance groups.

To enable direct comparison of the different deep learning methods, we implemented six methods in the discovery WSI LDLT cohort (**Figure 1A**). First, the AUC for the micro- and macro-averages was selected to evaluate model performance (**Supplementary Table S5**). Notably, the Light-GBM model had the highest AUC (0.978, 0.985 and 0.989) for the Normal, Rejection and Undefined groups in the training set (**Figure 1B**). Similar results were also validated in the testing set (**Supplementary Figure S1E**).

Surprisingly, in line with the critical role of lymphocytes in allograft rejection, allografts with greater lymphocyte infiltration in the portal vein (PV) areas had higher rejection prediction scores according to the DLP model (**Figure 1C**, **Supplementary Table S2**). Furthermore, allografts with a higher score for the histology features showed a significantly higher rejection prediction score, including the RAI score, G and S grading, bile stasis, biliary hyperplasia and fibrosis, but not hepatocyte degeneration (**Figure 1D**, **S1F**). Interestingly, the scores for histological features were significantly and positively associated with Rejection prediction scores, but negatively associated with Normal prediction scores and not associated with Undefined prediction scores (**Supplementary Figure S1G**, **Supplementary Table S6**).

To gain insight into how this DLP model predict allograft rejection, we explored the contribution of pathological signatures extracted from the model using LASSO and coefficient analysis (**Supplementary Figure S1H**). Of the 23 pathological labels extracted from the DLP model, 11 were significantly distinct lables among these three groups and were selected for subsequent analysis (**Supplementary Figure S2I**, **Supplementary Table S7**). It is worth noting that Prob054 had the highest value in the Rejection group, while Prob004 and Pred2 had the highest values in the Normal and Undefined groups, respectively (**Figure 1E**).

Next, we aimed to explore the clinical interpretability and applicability of the DLP model by linking pathological labels with the immune profile via a bulk RNA-seq analysis. The bulk RNA-serq cohort consists of paired transcriptomic and pathomic data derived from 18 LB after LDLT from pediatric patients receiving three types of Tacrolimus doses (**Supplementary Figure S1A**, **Supplementary Table S2**). Clustering analysis revealed that allografts with a high Prob054 value in the high-dose group exhibited high plasma cell (PC) and class-switched memory B cell infiltration compared to the low-dose and tolerance groups (**Figure 1F**). Conversely, allografts with high Preb2 and Prob004 values featured myeloid dendritic cells in the low-dose and tolerance groups (**Figure 1F**). Consistently, significant higher serum IgG level, but not IgA, IgM, IgG4, was found in the high-dose group, compared with those in the low dose and tolerance groups (**Supplementary Figure S1J**, **Supplementary Table S2**). KEGG analysis of up-regulated differential expression gene (DEG) revealed that the high-dose group was associated with an activated chemokine signaling pathway and Fc γ R-mediated phagocytosis (**Figure 1G**, **Supplementary Table S8**), while the low-dose and tolerance groups were associated with more haematopoietic cell lineages (**Figure 1H**, **Supplementary Table S8**). Our findings suggest that the DLP model is highly compatible with pathological risk factors in predicting allograft rejection, while the pathological label Prob054 could be a useful predictor of allograft rejection, possibly through an enhanced adaptive immune response.

### *In situ* identification and pathological roles of TLS in allograft rejection

**Figure 2:**
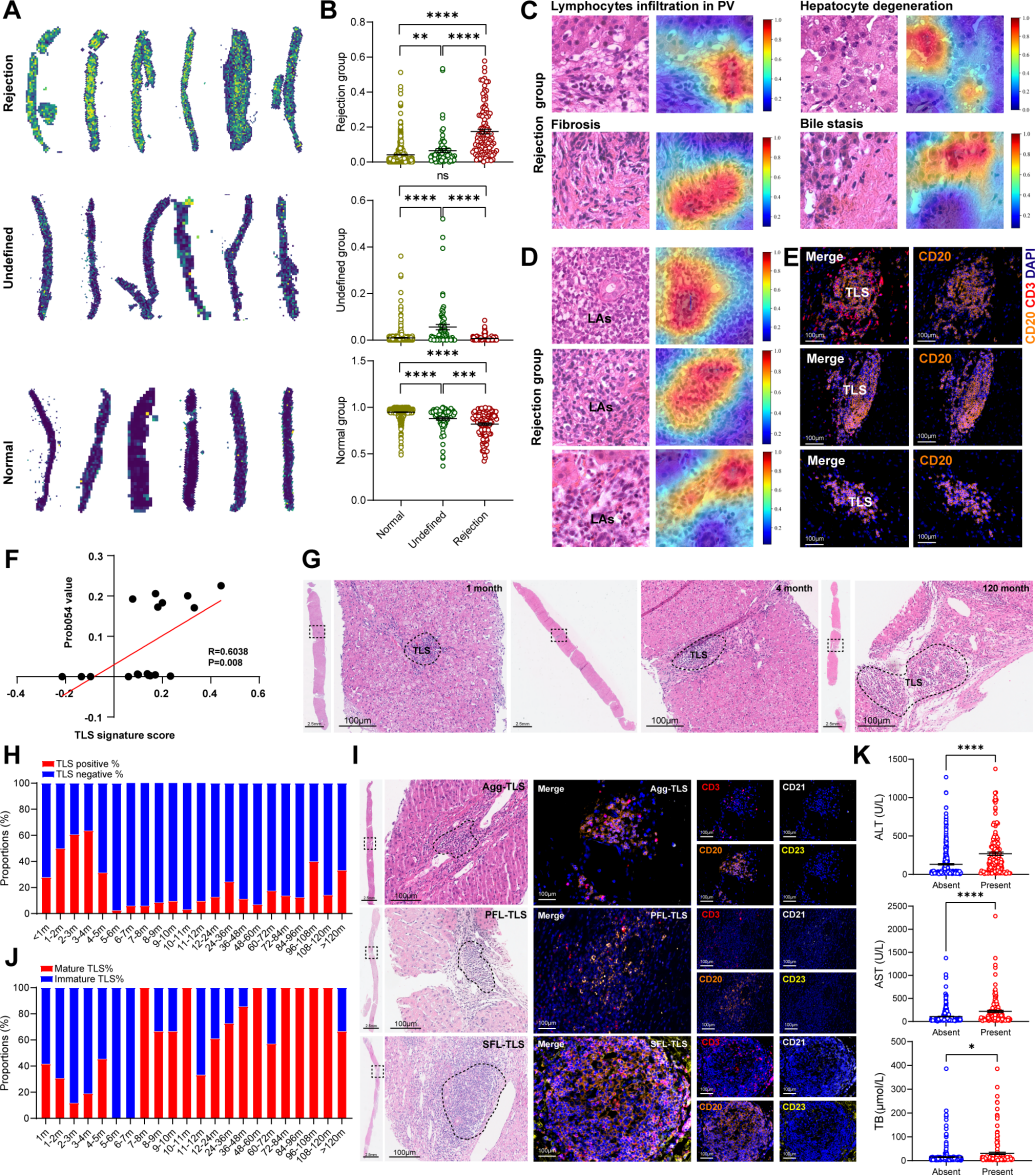
*In situ* identification and pathological roles of TLS in allograft rejection. **(A)** Representative prediction map for three allograft status mapping the predictive value of the respective tiles to their parent whole-slide images. **(B)** Dot plot showing prediction values of prediction scores of normal, rejection, and undefined allograft groups. **(C)** Visualization of the basis for predicting allograft rejection using GRAD-CAM on the most predictive tile, including lymphocyte infiltration in PV areas, fibrosis, bile stasis, and hepatocyte degeneration. **(D)** Representative patch images from WSI visualized using GRAD-CAM with lymphocyte aggregation. **(E)** Representative IHC staining of CD3 and CD20 for TLS identification in the PV areas. Scale bars, 100 μm. **(F)** Correlation analysis between TLS signature score and the values of Prob054, in the internal validation cohort of liver biopsy after pediatric LDLT. **(G)** Representative H&E staining images showing the presence of TLS at three time-points after LDLT (1 month, 4 month, and 120 month). Scale bars, 100 μm. **(H)** Bar chart depicting the relative frequency of patients without TLS (blue) and with TLS (red) at various time points after LDLT. **(I)** Representative H&E and mIHC staining images showing the presence of TLS with three stages after PLT (Agg-TLS, PFL-TLS and SFL-TLS). Scale bars, 100 μm. **(J)** Bar chart depicting the relative frequency of patients with immature TLS (blue) and mature TLS (red) at various time points after LDLT. **(K)** Dot plot showing the ALT, AST, and TB levels after LDLT according to the presence of TLS.

We further visualized the relevance of multi-class tissue prediction through probability heat maps obtained by the DLP model, with yellower and bluer colors indicating a higher and lower risk of rejection, respectively (**Figure 2A**). Meanwhile, the prediction scores demonstrated that the DLP model could accurately identify and visualize allograft stratification among Normal, Rejection, and Undefined status (**Figure 2B**). Our pathologists identified several distinctive features from these heatmaps through GRAD-CAM, including lymphocyte infiltration in the PV area, fibrosis, bile stasis and hepatocyte degeneration, where fibrosis and lymphocyte infiltration indicated hepatocyte damage and suggested a high risk of rejection (**Figure 2C**). Interestingly, hepatic sinusoid areas were consistently associated with normal allografts (**Supplementary Figure S2A**), despite the PV area being known for its role in promoting rejection and often being identified as a high-risk region by our network. Meanwhile, the most predictive tiles and visualizations in the Undefined group showed mild fibrosis and simplified bile stasis (**Supplementary Figure S2B**).

Notably, lymphocyte aggregates were mainly found in the perivascular areas, specifically in the red regions indicating a high risk of allograft rejection, suggesting that lymphocyte aggregates play a critical role in promoting allograft rejection (**Figure 2D**). Surprisingly, all of these lymphocyte aggregates detected using H&E staining were confirmed as TLS based on mIHC using CD3 and CD20 staining (**Figure 2E**). Meanwhile, Prob054 values were found to be significantly and positively associated with TLS signature scores (**Figure 2F**), while those of Preb2 and Prob004 were found to be negatively associated (**Supplementary Figure S2C**). Consistently, in line with TLS-associated genes (*CXCL9*, *CXCL10* and *CXCL11*) encoding chemokines, which were also identified as being associated with rejection in pan-organs (23), the chemokine signaling pathway was also activated in the high Tacrolimus dose group (allograft rejection group) (**Figure 1G**).

Notably, TLS were found to be prevalent in 19.5% (165/847) of LBs, almost in all clinically relevant allograft rejection cases, and reaching the highest proportion in biopsy samples taken 3–4 months after LDLT (**Figures 2G, H**). Meanwhile, three stages of TLS were identified in allograft biopsies based on CD20, CD21 and CD23 staining (**Figure 2I**). Meanwhile, the proportion of mature TLS showed a gradual upward trend after LDLT (**Figure 2J**). Remarkably, the presence of TLS had a significant influence on liver function (**Figure 2K**). Furthermore, when the allografts were divided according to TLS status, those harboring mature or immature TLS presented significantly higher levels of AST, ALT and TB compared to those without TLS (**Supplementary Figure S2D**). Similar results were further validated according to three TLS types (**Supplementary Figure S2E**).

Sensitivity analyses of recipients consistently showed that allografts diagnosed with biopsy-proven mild TCMR/ACR (**Supplementary Table S2**) were characterized by Agg-TLS, whereas most patients with antibody-mediated rejection (AMR) and chronic rejection (CR) were characterized by PFL-TLS and SFL-TLS, respectively (**Supplementary Figure S2F**). Specifically, allografts with TLS predicted a significant higher risk in rejection (**Supplementary Figure S2G**). Furthermore, the subtype of TLS also enabled a similar allograft rejection discrimination for LDLT patients (**Supplementary Figures S2H, I**). Meanwhile, we found that TLS demonstrated consistently high performance in the discovery LDLT WSI cohort (n=847) and the internal validation cohort (n=117) (**Supplementary Figures S2J, K**). Together, these results support the theory that TLS may act as a pathological promoter in regard to liver injury caused by allograft rejection.

### Targeting TLS formation alleviates allograft rejection by reducing ADCP effect mediated liver injury

**Figure 3:**
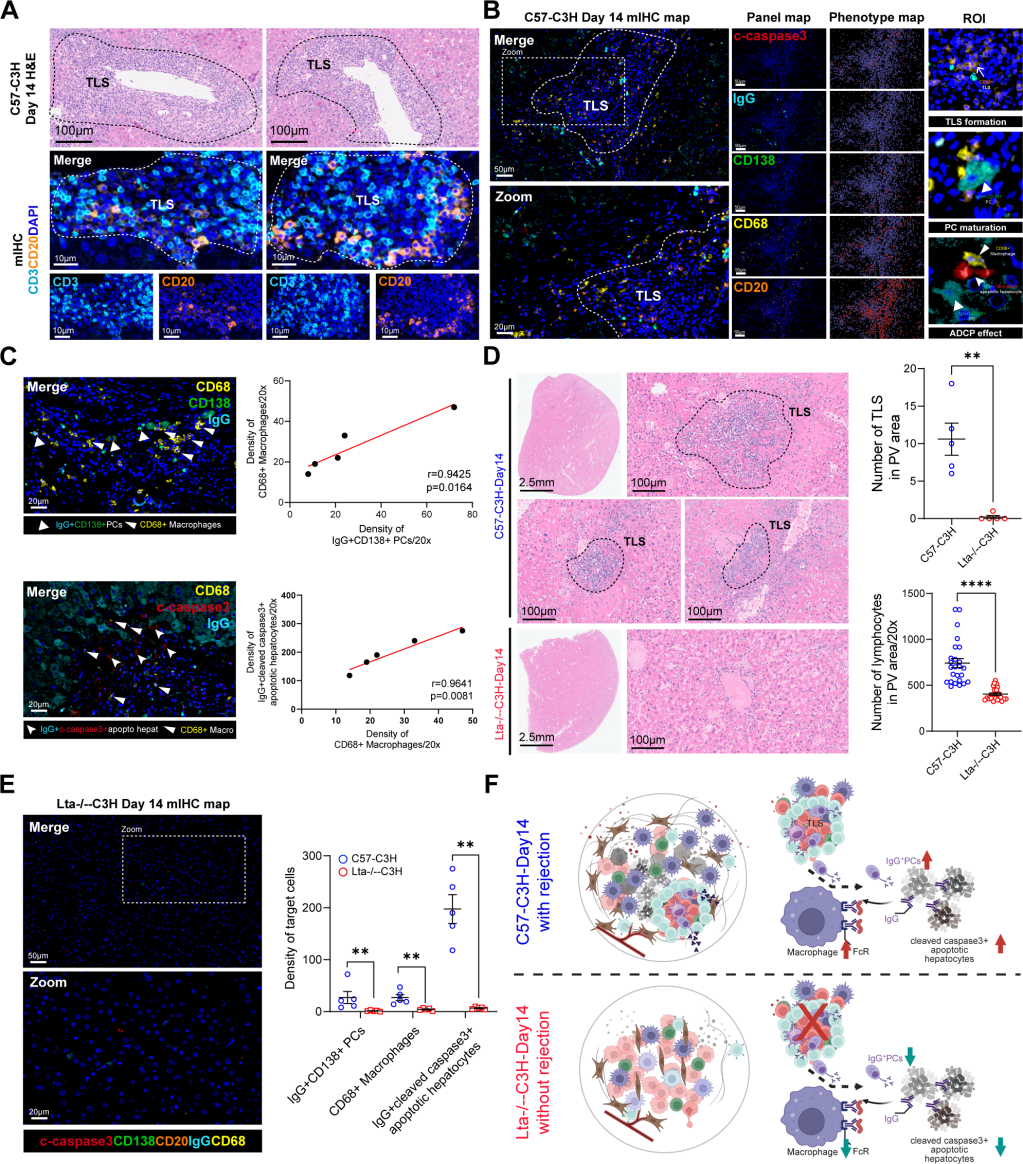
Targeting TLS formation alleviates allograft rejection by reducing ADCP effect mediated liver injury. **(A)** Representative H&E and mIHC staining images showing lymphocyte infiltration and aggregated B/T cells at day 14 in the C57 (donor) to C3H (receptor) MOLT model (n=5). Scale bars, 100 μm and 10 μm. **(B)** Representative mIHC staining images showing immune profile of CD20^+^ B cells, CD68^+^ macrophages, IgG^+^CD138^+^ PCs, and IgG^+^cleaved caspase-3^+^ apoptotic hepatocytes at day 14 in the C57 (donor) to C3H (receptor) MOLT model. Single panel map, corresponding phenotypic map based on the Gaussian weighted density map analysis, and region of interest (ROI) areas (including TLS formation, PC maturation, and IgG initiated macrophage related ADCP effect) were also demonstrated. Scale bars, 50 μm and 20 μm. **(C)** Representative mIHC staining images showing the spatial distribution feature and association between IgG^+^CD138^+^ PCs and CD68^+^ macrophages, as well as between CD68^+^ macrophages and IgG^+^cleaved caspase-3^+^ apoptotic hepatocytes. **(D)** Representative H&E staining images showing the comparison of TLS and lymphocyte infiltration (five PV areas were randomly selected from each sample) number between Lta^-/-^ (donor) to C3H (receptor) (n=5) and C57 mice (n=5). Scale bars, 100 μm. **(E)** Representative mIHC staining images and dot plot showing immune profile of CD20^+^ B cells, CD68^+^ macrophages, IgG^+^CD138^+^ PCs, and IgG^+^ cleaved caspase-3^+^ apoptotic hepatocytes at day 14 in the Lta^-/-^ (donor) to C3H (receptor) MOLT model. **(F)** Graphic abstract showing the schematic diagram of distinct immune profile, including TLS formation, PC maturation, and IgG initiated macrophage related ADCP effect between C57 (donor) to C3H (receptor) with rejection and Lta^-/-^ (donor) to C3H (receptor) without rejection after MOLT for 14 days.

Consistently, in line with the findings observed in the clinical cohorts, we found that B and T cell aggregation and TLS formation were formed after 2 weeks of the mouse orthotopic LT (MOLT) model based on paired H&E and mIHC staining, in the C57 (donor)-C3H (receptor) group (**Figure 3A**). Furthermore, consistent with the activated Fc γ R-mediated phagocytosis signaling pathway in the high-dose group (**Figure 1G**), we hypothesized that the antibody dependent cellular phagocytosis (ADCP) effect may play a role in liver injury of allograft rejection (24, 25). Notably, IgG^+^CD138^+^ PCs located outside of TLS in the allograft rejection sample were found to be in close proximity to CD68^+^ macrophages and IgG^+^cleaved caspase-3^+^ apoptotic hepatocytes (**Figure 3B**). Meanwhile, the density of IgG^+^CD138^+^ PCs was positively and significantly correlated with the density of CD68^+^ macrophages, as well as between CD68^+^ macrophages and IgG^+^cleaved caspase-3^+^ apoptotic hepatocytes (**Figure 3C**).

As a classic lymphotoxin ligand, LTA initiates TLS formation by triggering LTβR signaling, accelerating TLS niche maturation and maintaining TLS function (26). Consistent with the effect of B cell depletion in LT, the Lta^-/-^ group showed significantly reduced TLS formation and lymphocytic infiltration in the PV areas compared with the WT group after 2 weeks of MOLT (**Figure 3D**). Furthermore, the Lta^-/-^ group showed significantly less density of IgG^+^CD138^+^ PCs, CD68^+^ macrophages and IgG^+^cleaved caspase-3^+^ apoptotic hepatocytes (**Figure 3E**). Consequently, our study identified *LTA* as a promising target for treating TLS-mediated rejection by inhibiting LTA-induced TLS formation and reducing ADCP effect in the preclinical MOLT model (**Figure 3F**).

PTLF is a maladaptive repair process occurring in response to PTLR and liver injury, characterized by excessive deposition of extracellular matrix that significantly challenges the long-term success of LDLT (27). However, the driver factor induced PTLR to PTLF remained insufficiently explored.

To elucidate the potential pathological role of TLS in the progression of PTLR to PTLF, we enrolled a series of public transcriptomic datasets from allograft liver with biopsy-proven PTLR (GSE145780) and PTLF (GSE193135) (**Supplementary Figure S1A**). Notably, PTLR groups exhibited enriched immune profiles, demonstrating significantly higher scores of TLS signatures, B cell lineages, macrophages, and fibroblasts, compared with the those in the normal group (**Supplementary Figure S3A**). Similar results were also validated in the high Tacrolimus dose group (**Figure 1F**) and PTLF group (**Supplementary Figure S3B**). These results were further validated in clinical PTLR and PTLF allograft samples through mIHC staining, revealing a significantly higher number of B cell subsets (including CD138^+^ PCs, CD27^+^ Bm cells, and CD20^+^ B cells) in the PTLF group, compared with those in the PTLR and normal groups (**Supplementary Figures S3C, D**). Meanwhile, more TLS was also observed in the PTLF than those in the PTLR and normal groups (**Supplementary Figures S3E, F**).

To further explore the potential mechanism of TLS in the development of PTLR to PTLF, we identified 585 DEGs that were significantly up-regulated and associated with PTLR. Most of these DEGs were TLS-associated chemokines and antigen-presenting molecules (**Supplementary Figure S3G**), and markedly enriched in Epstein-Barr virus infection, cytokine-cytokine receptor interaction, and FcγR-mediated phagocytosis (**Supplementary Figure S3H**). Consistently, most of the up-regulated DEGs identified in the PTLF cohort were immunoglobulin genes, B-cell marker genes, and chemokines (**Supplementary Figure S3I**). Furthermore, similar molecular alterations associated with immune responses and TLS-related signaling pathways were also found in PTLF (**Supplementary Figure S3J**).

Taken together, these results emphasize the pathological role of enhanced TLS-dominated adaptive immunity in driving macrophage-associated ADCP effects via IgG, ultimately leading to hepatocyte injury and contributing the progression from PTLR to PTLF.

### Single cell analysis unveils the AtM B cells promoting TLS formation in allograft rejection

**Figure 4:**
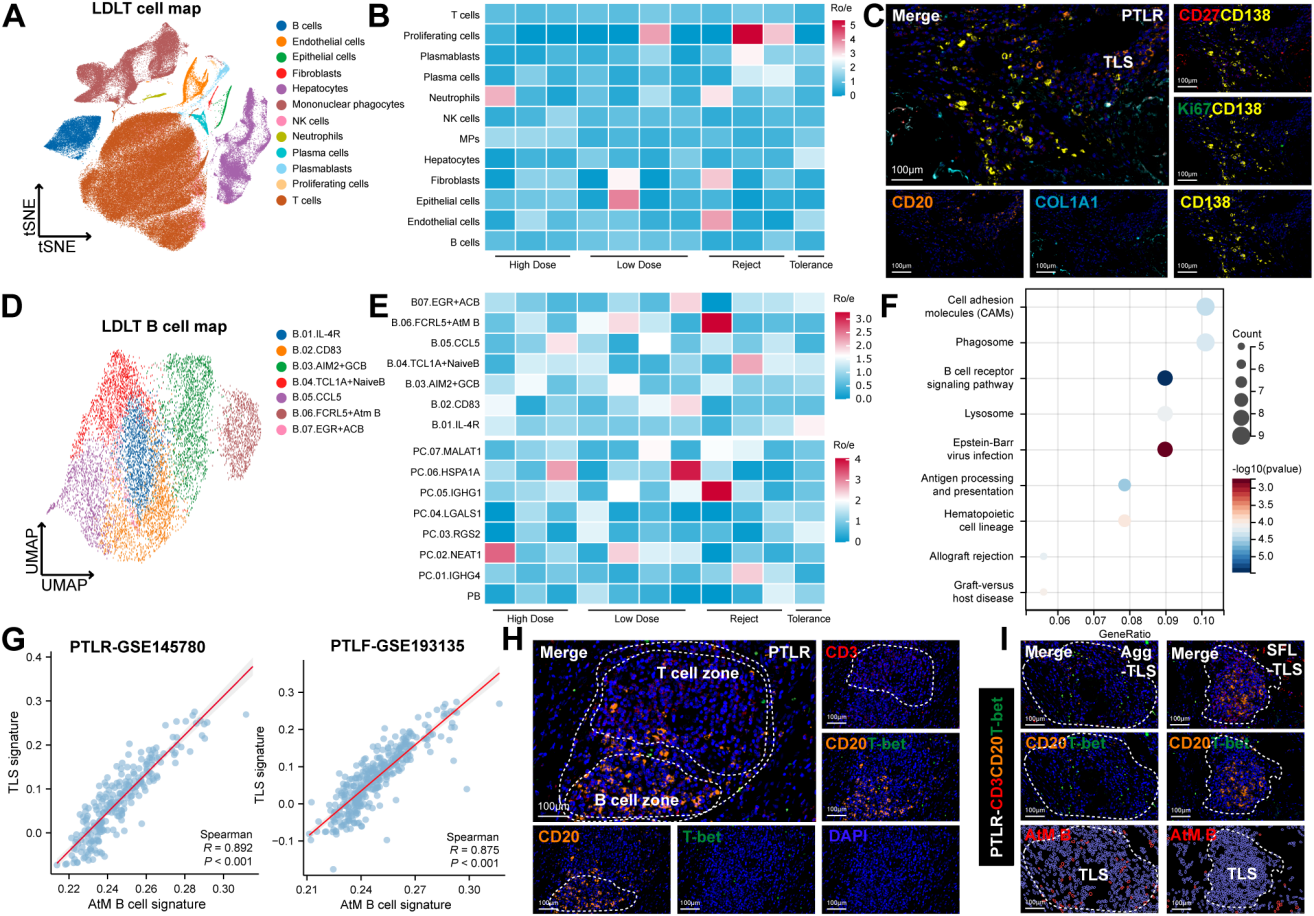
Single cell analysis unveils the AtM B cells promoting TLS formation in allograft rejection. **(A)** tSNE map depicting 12 main clusters found in 11 liver biopsies after LDLT. scRNA-seq data are integrated (high dose, n=3; low dose, n=4; rejection, n=3; tolerance, n=1). Each dot represents a cell, and each group of colored cells represents a different major cell type cluster. **(B)** Group preference of each cell clusters measured by the ratio of observed to randomly expected cell numbers (R_o/e_) calculated by the STARTRAC-dist algorithm. **(C)** Representative mIHC staining of differential abundance of Ki-67^+^CD138^+^ PBs, and CD27^+^CD138^+^ PCs in TLS areas of rejection group. Scale bars, 100 μm. **(D)** UMAP map depicting 7 clusters of B cells found in 11 liver biopsies after LDLT. **(E)** Ro/e analysis indicating group preference of each B cell clusters in four allograft groups after LDLT. **(F)** Bubble diagram depicting the signaling pathways enriched by KEGG analysis according to featured genes in AtM B cells. **(G)** Scatterplots showing gene expression of the TLS gene signature scores (39-gene) (y axis) with the difference of AtM B cell signature scores (x axis) in PTLR (GSE145780) and PTLF (GSE193135) datasets. **(H)** Representative mIHC staining of differential abundance and spatial distribution features of AtM B cells in the TLS areas in PTLR livers. Scale bars, 100 μm. **(I)** Representative mIHC staining of distribution features of AtM B cells between Agg-, and SFL-TLS in PTLR livers. Scale bars, 100 μm.

To investigate the formation mechanism of TLS in allograft rejection, we focused on these critical immune cell responses among four distinct immune status after LDLT (high dose, low dose, rejection, and tolerance; **Supplementary Table S2**). We identified 12 cell subsets through unsupervised clustering analysis by scRNA-seq performed in 11 liver biopsy samples (**Figures 4A**, **S4A**). Given the significant immune microenvironment heterogeneity in these four groups, Ro/e index analysis indicated the group preferences of distinct cell clusters, since plasmablasts (PBs), and PCs were enriched in the rejection group than those in other groups (**Figure 4B**), which was consistent with our previous findings (**Figure 1F**). The mIHC staining further confirmed the presence of Ki-67^+^CD138^+^PBs, and CD27^+^CD138^+^PCs around the TLS areas of rejection group (**Figure 4C**). The presence, location, and distribution trajectory of B cells and PCs implicated that TLS acted as a crucial niche for B cell differentiation or development within allograft rejection.

B cells are a major component of the liver microenvironment, where they are predominantly associated with TLS (28). To further explore the heterogeneity of B cells, we identified 7 distinct B cell and 8 antibody secreting cell (ASC) subsets (**Figures 4D**, **S4B**, **Supplementary Table S9**). Notably, Ro/e index analysis confirmed the heterogeneous distribution characteristics that AtM B cells and IGHG1^+^ PCs, IGHG4^+^ PCs were enriched in the rejection, high dose, and low dose groups, but depleted in the tolerance group (**Figure 4E**). In contrast, IL-4R^+^ B cells and RGS2^+^ PCs were significantly enriched in the tolerance group (**Figure 4E**). Featured genes based KEGG analysis indicated that AtM B cells were enriched with cell adhesion molecules (CAMs), B cell receptor signaling, EBV infection, antigen processing and presentation, and allograft rejection (**Figure 4F**), which was significant differed from IL-4R^+^ B cells (**Supplementary Figure S4C**).

Since the remarkable enrichment of both AtM B cells and TLS in allograft rejection, we hence examined their relationship by calculating the gene signature scores of AtM B cells and TLS. Notably, AtM B cells signature score was significantly and positively associated with TLS signature score in both PTLR and PTLF datasets (**Figure 4G**). Our mIHC staining results further confirmed that AtM B cells were located in the boundary of B/T cell zone of the TLS areas in both PTLR (**Figure 4H**) and PTLF livers (**Supplementary Figure S4D**). Consistent with the early EF response and facilitation of TLS formation (29), we observed that AtM B cells were predominantly located in the center of immature TLS (Agg-TLS), whereas migrated to the periphery of mature TLS (SFL-TLS) in both PTLR and PTLF (**Figures 4I**, **S4E**). Notably, significant less AtM B cells were detected within the core of SFL-TLS, compared with those in Agg-TLS in both PTLR and PTLF (**Supplementary Figure S4F**). Together, our results suggest the underlying cooperation of AtM B cells in the formation of TLS in both PTLR and PTLF.

### Exhausted CD8^+^ T_EM_ cells promote AtM B cells differentiation via IFN-γ

**Figure 5:**
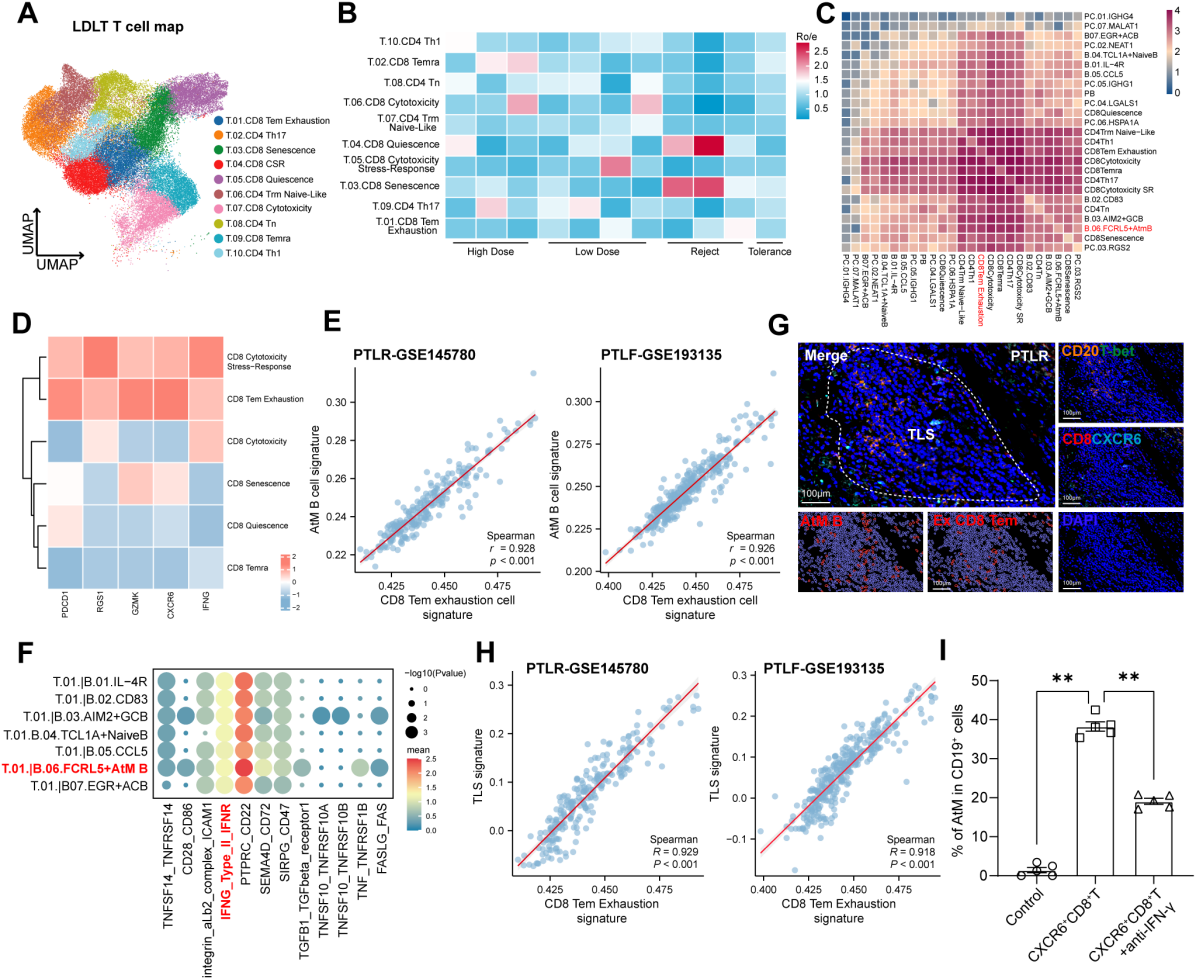
Exhausted CD8^+^ T_EM_ cells promote AtM B cells differentiation via IFN-γ. **(A)** UMAP map depicting 10 clusters of T cells found in 11 liver biopsies after LDLT. **(B)** R_o/e_ analysis indicating group preference of each T cell clusters in four allograft groups after LDLT. **(C)** Spearman correlation between B cell subsets and T cell subsets deconvoluted by scRNA-seq. **(D)** Heatmap showing the expression of *PDCD1*, *RGS1*, *GZMK*, *CXCR6*, and *IFNG* in CD8^+^ T cell subsets. **(E)** Scatterplots showing gene expression of the AtM B cell signature scores (y axis) with the difference of CD8 T_EM_ exhaustion cell signature (x axis) in PTLR (GSE145780) and PTLF (GSE193135) datasets. **(F)** Bubble heatmap showing the mean interaction strength between the neighbor clusters at the boundaries for ligand-receptor pairs through Cellphonedb analysis. Dot size indicated the statistical significances by permutation test. Dot color indicated the mean interaction strength levels. **(G)** Representative mIHC staining of distribution association between AtM B cells and exhausted CXCR6^+^CD8^+^ T_EM_ cells in PTLR livers. Scale bars, 100 μm. **(H)** Scatterplots showing gene expression of the TLS gene signature scores (y axis) with the difference of CD8 T_EM_ exhaustion cell signature (x axis) in PTLR (GSE145780) and PTLF (GSE193135) datasets. **(I)** Box plot showing proportions of AtM B cells in CD19^+^ B cells detected by flow cytometry analysis. Healthy blood CD19^+^ B cells were stimulated with alone as control, in the presence of CXCR6^+^CD8^+^ Tem cells, or anti-IFN-γ antibody combined with CXCR6^+^CD8^+^ Tem cells for 7 days.

In TLS, B cells engaged in intricate interactions with T cells to initiate adaptive immune responses (30). To evaluate the presence of T cell subsets and T-B cell interactions in distinct status of allografts, we further clustered 10 T cell subsets using scRNA-seq data (**Figures 5A**, **S5A**). Notably, Ro/e index analysis confirmed that exhausted CD8^+^ T effect memory (T_EM_) cells, CD8^+^ Senescence, and CD8^+^ Quiescence subsets were enriched in the rejection group, but depleted in the tolerance group (**Figure 5B**).

As mentioned above, the abundance of TLS, AtM B cells, and CD8^+^ T subsets in allograft rejection, highlighting their cell-cell interactions mainly localized inside the TLS. We thus correlated lineage-normalized cell-type frequencies across allografts to investigate their interactions. Notably, AtM B cells showed high correlation with all CD8^+^ T cell subsets at single cell level, especially with exhausted CD8^+^ T_EM_ cells (**Figure 5C**). Remarkably, exhausted CD8^+^ T_EM_ cells were featured with high expression of PDCD1, GZMK, CXCR6, and IFNG, exhibiting its exhausted phenotype (**Figure 5D**). Notably, GSVA analysis further validate the correlations of these cells observed in our scRNA-seq data, indicating that exhausted CD8 T_EM_ cells signature score was significantly and positively correlated with AtM B cell score in both PTLR and PTLF datasets (**Figure 5E**). Interestingly, we found that multiple ligand-receptor pairs associated with the differentiation and recruitment of B cells were highly expressed between exhausted CD8 T_EM_ cells and B cell subsets, especially with AtM B cells, including IFNG-Type II_IFNR and PTPRC-CD22 (**Figures 5F**, **S5B**). Remarkably, our mIHC staining analysis further showed exhausted CXCR6^+^CD8^+^ T_EM_ cells co-localized with AtM B cells in TLS, suggesting that their interaction directly (**Figure 5G**). Consistently, exhausted CD8^+^ T_EM_ cells signature score was significantly and positively associated with TLS signature score in both PTLR and PTLF datasets (**Figure 5H**).

As mentioned above, the abundance of IFNG expression of exhausted CD8 T_EM_ cells (**Figure 5D**) highlighted the potentially interaction between AtM B and exhausted CD8 T_EM_ cells through IFNG-Type II_IFNR pathway (**Figure 5F**). Flow cytometry further validated that CD19^+^CD11c^+^ AtM B cells were barely detected in the B cells cultured alone, but more efficiently with CXCR6^hi^CD8^+^ T cells, which was significantly attenuated by neutralizing IFN-γ (**Figures 5I**, **S5C**). These findings suggest that exhausted CD8 T_EM_ cells are crucial for the AtM B cell differentiation through IFN-γ and thus promoted TLS formation and allograft rejection.

### Target IFN-γ-JAK-STAT axis governs AtM B cell differentiation, TLS formation and alleviates allograft rejection

**Figure 6:**
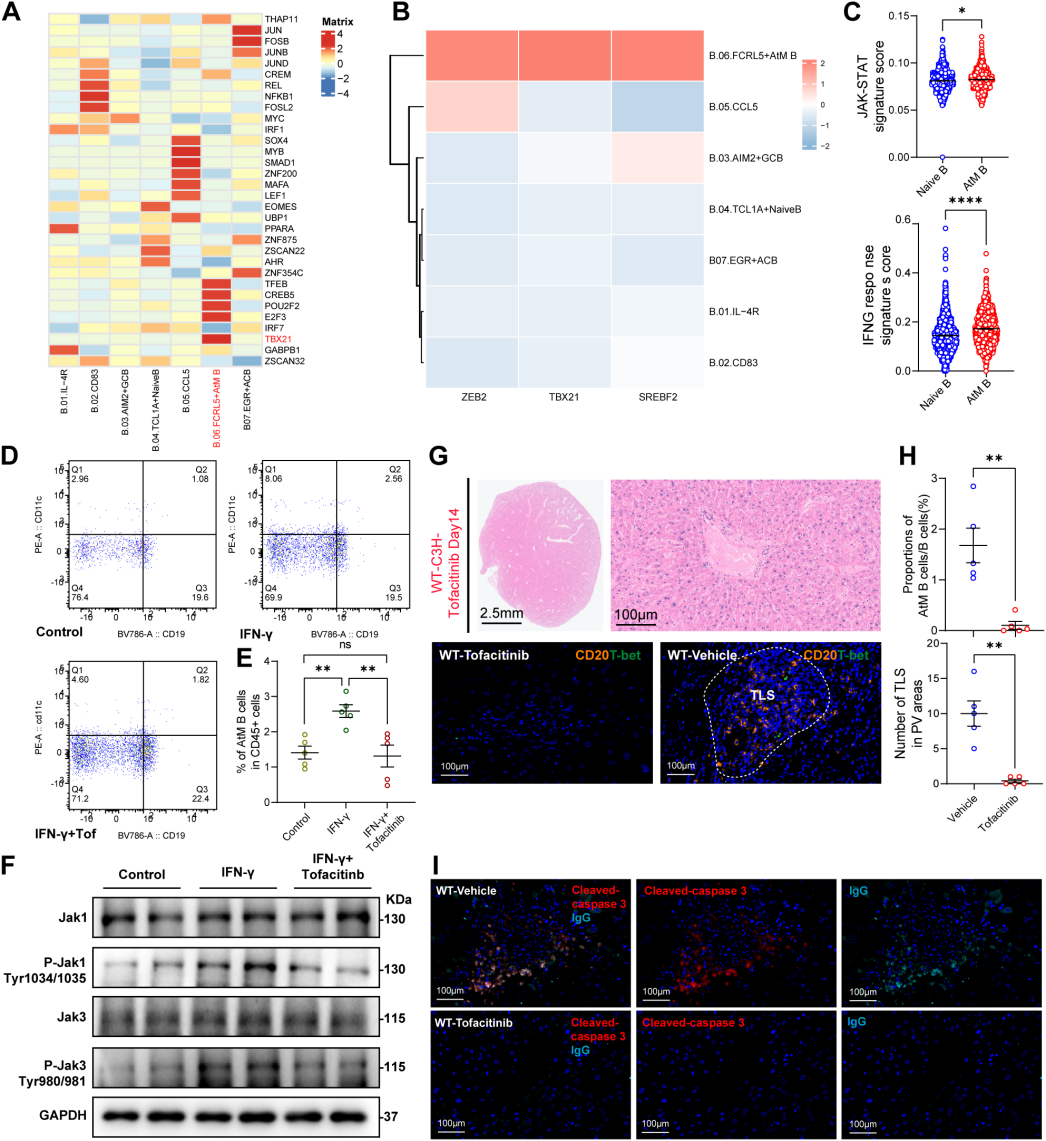
Target IFN-γ-JAK-STAT axis governs AtM B cell differentiation, TLS formation and alleviates allograft rejection. **(A)** Heatmap showing key regulatory TFs identified by RSS scoring through SCENIC analysis. **(B)** Heatmap showing the expression of *ZEB2*, *TBX21*, and *SREBF2* in seven B cell subsets. **(C)** Dot plot showing the comparison of JAK-STAT and IFNG response signature scores between TCLA_ Naive B cells (blue) and AtM B cells (red). **(D)** Representative flow cytometry plots of AtM B cells within three groups (control, IFN-γ, and IFN-γ combined with Tofacitinib). **(E)** Dot plot showing proportions of AtM B cells in CD45^+^ cells. Healthy blood B cells were stimulated with alone, in the presence of IFN-γ, or IFN-γ combined with Tofacitinib for 4 days. **(F)** Representative images of western blot analysis for the protein levels of JAK-STAT pathway in B cells after co-cultured with medium from with and without IFN-γ treatment and Tofacitinib. **(G)** Representative H&E staining images in mice treated with Tofacitinib after MOLT for 14 days and mIHC staining images of AtM B cells in mice treated with and without Tofacitinib after MOLT. Scale bars, 100 μm. **(H)** Dot plot showing proportions of AtM B cells within B cells and number of TLS in the PV areas in mice treated with and without Tofacitinib after MOLT. **(I)** Representative mIHC staining images showing IgG^+^ cells and cleaved caspase-3^+^ apoptotic hepatocytes at day 14 in mice treated with and without Tofacitinib after MOLT (C57 (donor) to C3H (receptor)). Scale bars, 100 μm.

Transcription factors (TFs) regulate various developmental and functional aspects of B cells (31). To better define the differentiation of AtM B cells, we investigated top 5 key regulatory TFs, identified by RSS scoring based on SCENIC analysis, suggesting that *TBX21* (encoding T-bet) as a key regulon of AtM B cells (**Figures 6A**, **S6A, B**). Notably, AtM B cells were also featured with the high expression of *ZEB2*, *TBX21*, and *SREBF2* (**Figure 6B**), consistent with specific TFs of age-associated B cells (ABCs) in autoimmune diseases (31). Remarkably, the high concordance in featured genes was observed between ABCs and AtM B cells (**Supplementary Figure S6C**, **Supplementary Table S10**), demonstrating the conserved patterns of these B cells that underpins chronic tissue injury.

We further investigated the signaling pathways by *TBX21* influenced AtM B cells differentiation. Notably, JAK-STAT and IFNG response signature scores were significant up-regulated in AtM B cells, compared with those in TCLA_ Naive B cells (**Figure 6C**), and significant enriched in PTLR (**Supplementary Figure S3H**). Remarkably, *in vitro* simulation with IFN-γ significant up-regulated AtM B cells differentiation (**Figures 6D, E**) and JAK-STAT signaling pathway (**Figure 6F**), which was significantly attenuated by the JAK-STAT inhibitor-Tofacitinib (**Figures 6D–F**).

Tofacitinib had proven effective in dampening JAK/STAT signaling (32), while long-term application of Tofacitinib continued to be effective in preventing renal allograft acute rejection and preserving renal function (33). We therefore tested their effects on AtM B cells within MOLT model. Notably, Tofacitinib significantly reduced AtM B cells accumulation, as well as TLS formation in the PV areas (**Figures 6G, H**), as well as reduced hepatic IgG accumulation, and less apoptotic hepatocytes (**Figures 6I**, **S6D**) at 2 week after MOLT.

Thus, targeting the JAK-STAT pathway can block AtM B cells differentiation and TLS formation in MOLT model, highlighting Tofacitinib as a promising drug for the treatment of AtM B cells and TLS-mediated hepatocyte injury in allograft rejection.

## Discussion

LT is the definitive cure for end-stage liver disease, while acute or chronic rejection and fibrosis remain major barriers to long-term allograft survival. Although immunosuppressive drugs can alleviate rejection following LT, the long-term side effects of the medication continue to pose significant challenges to patients’ quality of life. Therefore, it is essential to identify the key immune cells and targets and to regulate the immune system in order to manage allograft rejection and fibrosis. Compared to adult LT, which is characterized by a variety of factors such as mixed backgrounds, pediatric LDLT has many advantages, including a single disease type, younger donor age, higher donor liver quality, and shorter cold ischemia time, making it more conducive to systematic research on allograft rejection. Hence, our study provides an integrated, multi-dimensional atlas of TLS promoting TLS-orchestrated B-cell adaptive immunity drives hepatocyte injury in allograft rejection based on pediatric LDLT cohorts. Using an integrated DLP model, clinical cohorts, scRNA-seq and mIHC profiling, we identify the pathological role of TLS in allograft rejection and fibrosis. We demonstrate that exhausted CD8^+^ Tem cells promote AtM B cell differentiation via IFN-γ, thereby inducing TLS formation. This results in the maturation of IgG^+^ PCs and hepatic IgG accumulation, which initiates ADCP of apoptotic hepatocytes by CD68^+^ macrophages. Furthermore, *in vivo* inhibition of JAK1/3 restricts AtM B cell differentiation, TLS formation, IgG production and hepatocyte injury, which could inform the development of novel therapeutics for rejection pathology.

Deep learning tools can analyse the wide spectrum of data types, dynamics and interrelationships among clinical, laboratory, pathological, imaging and omics data from both donor and recipient in the post-transplant, providing diagnostic and prognostic value. For predicting short- and long-term patient survival, random forest (RF) and feed-forward neural network (NN) achieved AUC values of 0.771 and 0.703 (34). For predicting short- and long-term graft failure, the ANN, recurrent NN and RF models achieved AUC values of 0.96, 0.78 and 0.818 (34). However, only the ANN approach was performed to predict acute allograft rejection in LT recipients using routine laboratory data, with the AUC of 0.902 (34). Notably, we applied six types of DL approaches and found that the LightGBM-DLP model performed best in multi-class tissue classification. The performance varying depending on the patient population (adult vs. pediatric), whether the study was single-centre or multicentre, the size of the dataset, the type of DL tool used and whether the data were longitudinal or static at the time of transplant.

TLS is organized clusters of immune cells that develop in non-lymphoid tissues under specific conditions, such as autoimmunity, chronic infections and cancers (35), In organ transplantation, TLS plays a complex and multifaceted roles. Of 319 (35) murine allograft heart transplants, 78 revealed the presence of TLS, with 78% of allografts undergoing chronic rejection (7). Conversely, TLS was also associated with immune tolerance (36). Remarkably, TLS was observed in transplants after 6–10 weeks post-transplantation, even in the absence of acute rejection episodes (37). Meanwhile, the absence of intra-tumoral TLS was associated with a worse prognosis and mTOR signaling activation in hepatocellular carcinoma with LT (38). This heterogeneity, or even the opposite effect, may be due to differences in disease models, organ contexts, observation periods and treatment approaches. Then, the specific characteristics and dynamics of TLS in pediatric LDLT recipients may differ due to factors such as the developing immune system of children, underlying liver disease etiology, and immunosuppressive regimens used in this population. Although several studies have reported associations between TLS and antibody-mediated rejection or chronic allograft dysfunction in other organs (7, 37), the specific contribution of TLS to the pathophysiology of allograft rejection after pediatric LDLT remains poorly defined. Notably, our findings indicated that high PCs and class-switched memory B cell were enriched in the high IS dose group, and the proportion of mature TLS showed a gradual upward trend after LDLT, indicating that TLS related B cells and adaptive immune response was abnormal activated in the allograft rejection, potentially synchronized with the progression of PTLR to PTLF. Meanwhile, abnormal activation of B cells and adaptive immune response in allograft rejection was further validated in adult LT datasets, suggesting that TLS function is consistent in allograft rejection for both pediatric and adult patients.

The progression from PTLR to PTLF is no longer considered an oversimplified description of scarring caused by chronic hepatitis. It is now understood to be a cascade network advancing from antibody-complement-microvascular injury to IL-33/TGF-β/YAP multi-axis synergy. This ultimately results in the epigenetic activation of hepatic stellate cells (HSCs) and persistent collagen deposition. However, TLS is defined as a niche that drives inflammation and aggregates fibroblasts and immune cells, thereby remodelling the local immune microenvironment and enhancing adaptive immunity. In order to elucidate the potential pathological role of TLS in the progression from PTLR to PTLF, two public transcriptomic datasets were enrolled from allograft livers with biopsy-proven PTLR (GSE145780), and the other comprising livers with biopsy-proven PTLF (GSE193135). Notably, significantly increased TLS signatures were found in both the PTLR and PTLF groups, compared with the control groups. Furthermore, more TLS was also observed in PTLF than in PTLR and normal groups based on mIHC staining. Meanwhile, analysis of the potential mechanisms highlighted that FcγR-mediated phagocytosis may play a pathological role in hepatocyte injury and contribute to the progression from PTLR to PTLF.

The liver is conventionally viewed as an immunologically “privileged” organ because of its unique micro-vascularisation, large population of tolerogenic Kupffer cells (KCs) and low-level expression of HLA class II (38). During the past three years our understanding of the immune-inflammatory networks that operate after LT has been re-shaped by scRNA-seq, spatial transcriptomics and high-parameter cytometry applied to both protocol and for-cause biopsies. Single-cell atlases revealed that LDLR^+^ activated MDSC prevented allograft rejection (39), while the inflamed NK cells, CD14^+^RNASE2^+^ monocytes, and FOS^+^ monocytes were emerged as predictive indicators of ACR (40). However, limited data had been presented in the exploration of B cells in LT. Enhanced B cell presentation of donor antigen relative to HLA-nonidentical antigen in a novel cell-based assay and with a downregulated HLA-DOA gene in children (41). QuSAGE analysis showed that the expression of genes in the PC clusters related to protein secretion, while the B cell clusters related to interferon response pathways (42). Consistent with bulk RNA-seq results, we also explored the immune landscape within different immune status and IS doses through scRNA-seq, especially for B cells. Notably, PC and PB were enriched in rejection allografts. To the best of our knowledge, this is the largest study aimed to systematically identified B cell subsets in LDLT. Moreover, we investigated the role of hepatic AtM B cells derived from vascular-surrounding TLS *in situ* in the liver, where AtM B cells differentiated and promoted pathogenical TLS formation. However, the precise mechanism by which AtM B cells develop into PC cells through the EF response awaits further exploration in the future.

Significant cross-talk between T cell subsets and AtM B cells were found in viral infection (43), and lupus nephritis (44). In line with the substantial increased of CD8^+^ tissue-resident memory T cells (TRMs) in allograft rejection (45), we demonstrated that these PD-1^+^CXCR6^+^CD8^+^ exhausted T_EM_ cell potentially promoted AtM B cells differentiation via IFN-γ secretion, mainly through the JAK-STAT signaling pathway. Total B cell depletion using anti-CD20 monoclonal antibody was found to rapidly and effectively reducing the number of circulating and allograft-resident memory B cells and PBs, as well as inhibiting donor-specific antibody (DSA) production and significantly lowers the incidence of AMR (46). Remarkably, B cell deficiency simultaneously eliminated Bregs with immunoregulatory functions, resulting in diminished T-cell suppression (47). Thus, identifying the key B-cell subsets and targets involved could significantly reduce the adverse effects associated with extensive B-cell depletion.

IFN-γ is a key driving factor in the differentiation of AtM B cells (29), and is markedly increased in allograft recipients (48). Meanwhile, IFN-γ is required for the expression of T-bet (encoded by *TBX21*) via the JAK-STAT pathway in chronic lymphocytic leukaemia cells (49). Consistently, the transcription factor T-bet has been shown to regulate the maintenance and differentiation potential of lymph node and effector memory B cell subsets (50). Notably, we found that TBX21 is a key regulator of AtM B cells, and that IFN-γ significantly increases AtM B cell differentiation and JAK-STAT signaling, which is significantly attenuated by the JAK1/3 inhibitor tofacitinib. In line with long-term tofacitinib continuing to be effective in preventing renal allograft rejection and preserving renal function (33), we infer that tofacitinib may be a promising therapeutic agent for preventing allograft rejection after LDLT. However, further investigation is required due to the risk of persistent serious infection.

However, there are several limitations to our study. Firstly, our training data was sourced from a single institution with potential selection bias. However, more external validation cohorts from other pediatric LDLT centers should be included to further validate the stability and reliability of TLS in allograft rejection. Secondly, as the patients in both cohorts were enrolled from retrospective cohorts, resulting in the absence of donor clinical information and paired donor-specific antibody (DSA) level at the time of LB, while more detailed clinical data needs to be collected in future clinical cohorts. Thirdly, the heterogeneity of transcriptomic cohorts warrants further consideration. Fourthly, the MOLT model is a vital tool for investigating transplant immunology, ischaemia-reperfusion injury and postoperative regeneration. However, translating its findings into clinical practice has repeatedly been thwarted by multiple interspecies differences, including anatomical scale and haemodynamics, immunological landscape, and metabolic and regenerative rhythms. The translation of MOLT results to clinical applications requires more research support, especially in the terms of the translatability of findings from mice to pediatric LDLT patients. Lastly, although TLS presence and classification are carefully analyzed, this study does not evaluate TLS density, which has been shown in other cancer and transplant contexts to have prognostic implications.

## Data Availability

scRNA-seq sequencing dataset is available at the National Genomics Data Center (accession no. PRJCA050696). Any additional information required to reanalyze the data reported in this work paper is available from the lead contact upon request.
