# Supplementary material for: Tertiary lymphoid structure drives allograft rejection via IFN-γ-JAK-STAT-dependent atypical memory B cell differentiation
Source: Front Immunol. 2025 Dec 11;16:1728290. doi: 10.3389/fimmu.2025.1728290 (PMC12738316; doi:10.3389/fimmu.2025.1728290)
Supplement: Supplementary file 1 [file Table1.docx]

**Supplemental Figures and Figure legends**

**Tertiary lymphoid structure drive allograft rejection via IFN-γ-JAK-STAT-dependent atypical memory B cell differentiation**

**
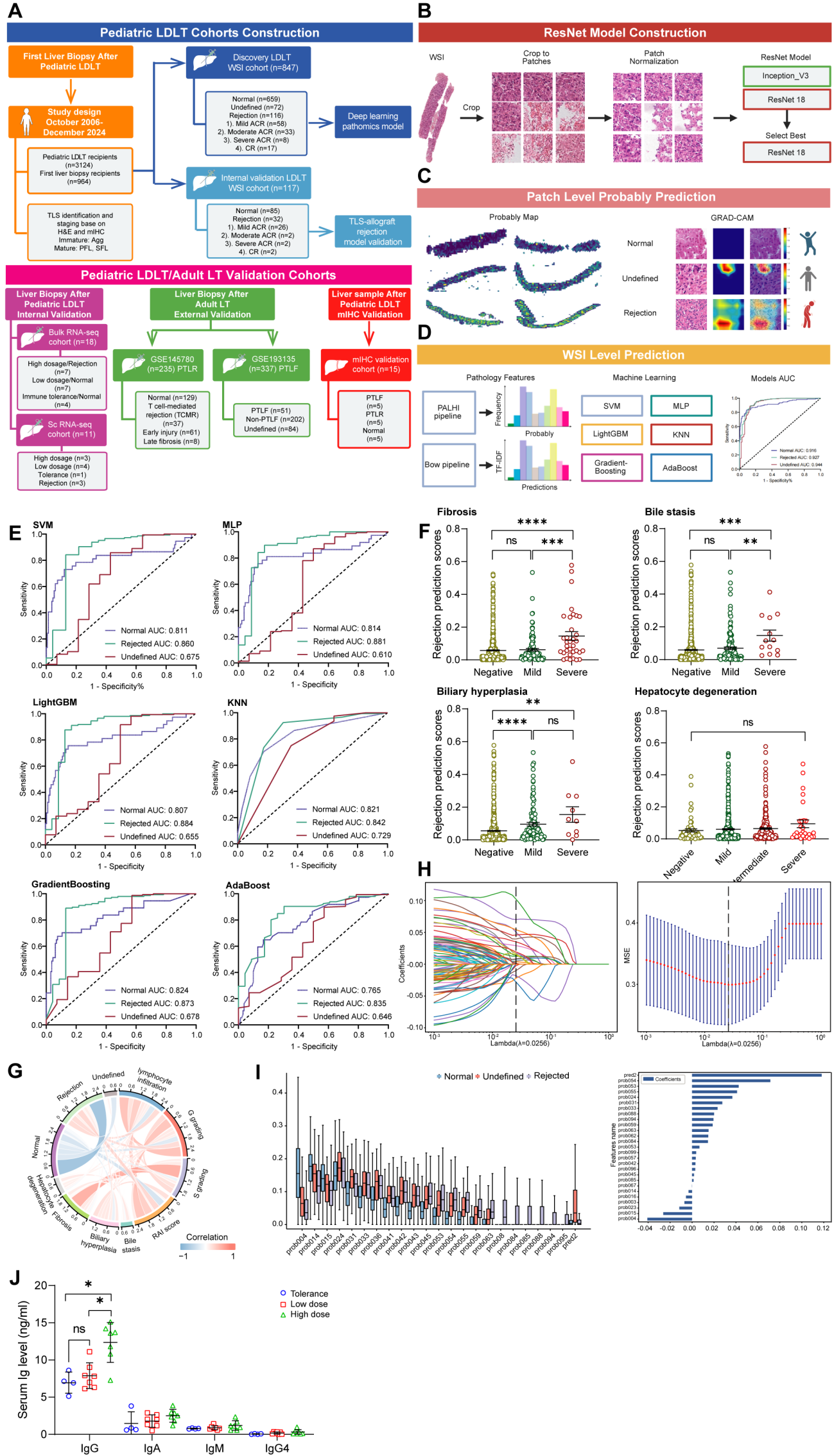
**

**Figure S1. Develop an interpretable DLP model to predict allograft rejection**

(**A**).Overview of the study design and patient recruitment strategy for liver transplantation cohort construction. (**B**).Flowchart for constructing a machine learning model based on pathological WSIs, including WSI segmentation, color standardization, and deep learning model construction. (**C**).Patch-level prediction. Re-integration of the patch level into WSIs: Statistics are performed on the prediction results of all the patches partitioned into a WSI, and Probably Map and GRAD-CAM are used for visualization. (**D**).WSI level prediction. The PALHI and BoW pipelines integrate multiple patch-level likelihoods into a WSI-level prediction. Machine learning model construction: The histogram features and TF-IDF features were pre-merged and classified using different predictions (normal, rejected, and undefined). (**E**).Receiver operating characteristic curves of six deep learning methods for assessing allograft immune status, including Normal (purple line), Rejected (green line) , and Undefined (red line) in the testing set. (**F**).Dot plot showing rejection prediction score shows a progressive increase from negative to severe based on fibrosis, bile stasis, and biliary hyperplasia, but not in hepatocyte degeneration. (**G**).Chord diagrams visualized pairwise correlation analysis among allograft status, and pathological features, including lymphocytes infiltration in PV areas, fibrosis, bile stasis, and hepatocyte degeneration in the discovery LDLT WSI cohort. (**H**).Variable trajectory after LASSO screening following fusion of histogram features and TF-IDF features. LASSO coefficient screening chart. Weight coefficient diagram of features selected by LASSO. (**I**).Box plot depicting values of predicted pathological features among three allograft groups. (**J**).Dot plot showing serum IgG, IgA, IgM, IgG4 levels after LDLT according to three dose of IS.

**
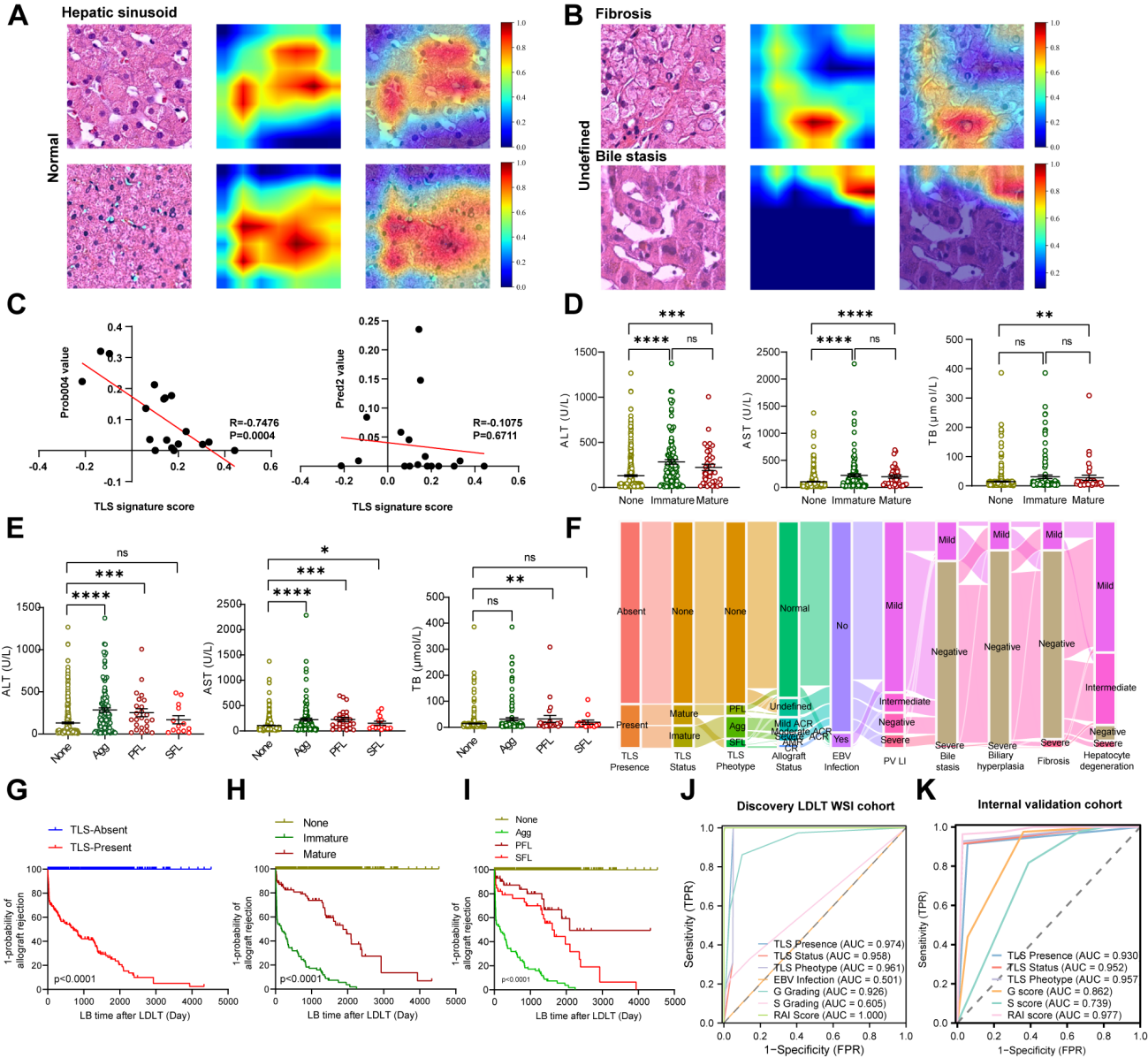
Figure S2. *In situ* identification and pathological roles of TLS in allograft rejection**

(**A**).Representative patch images from WSI visualized by GRAD-CAM with hepatic sinusoid in normal groups. (**B**).Representative patch images from WSI visualized by GRAD-CAM with fibrosis and bile stasis in undefined groups. (**C**).Correlation analysis between TLS signature score and the values of Prob004 and Pred2, respectively, in the bulk-RNA-seq cohort of liver biopsy after LDLT. (**D**).Dot plot showing ALT, AST, and TB levels after LDLT according to the maturation of TLS. (**E**).Dot plot showing ALT, AST, and TB levels after PLT according to three stages of TLS. (**F**).Mulberry tree diagram showing the association between TLS (presence, status, and phenotype) and allograft status (EBV infection and pathological features). (**G-I**).Kaplan-Meier survival curves for allograft rejection based on (**G**) TLS presence (absent, present); (**H**) TLS status (None, immature, and mature-TLS); (**I**) TLS stages (None, Agg, PFL, and SFL) in the discovery LDLT WSI cohort. (**J-K**).Receiver operating characteristic curves of TLS (TLS presence, TLS status, and TLS phenotype) and allograft status (G grading, S grading and RAI score) in the (**J**) discovery LDLT WSI cohort (n=847)and (**K**) internal validation cohort (n=117).

**
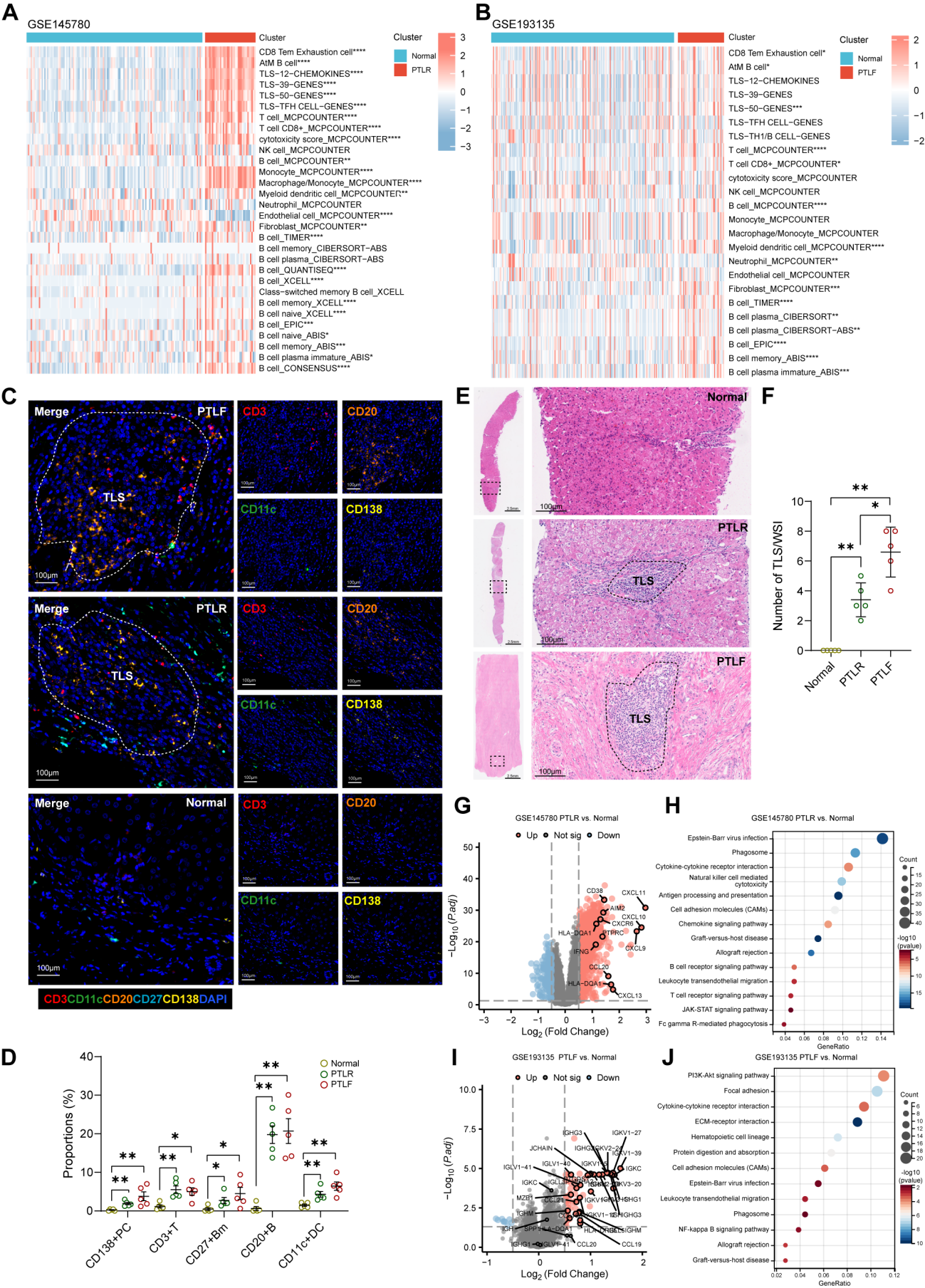
Figure S3. Targeting TLS formation alleviates allograft rejection by reducing ADCP effect mediated liver injury**

(**A-B**).Heat map showing multiple TLS signature scores, immune infiltration (MCP counter) and B cells (MCP counter, TIMER, CIBERSORT, QUANTISEQ, XCELL, ABIS, and CONSENSUS) (**A**) between PTLR and normal groups (GSE145780) and (**B**) between PTLF and normal groups (GSE193135). (**C**).Representative mIHC staining of differential abundance of B cells, T cells, PCs, and myeloid dendritic cells in PTLF, PTLR, and normal groups. (**D**).Dot plot showing the proportions of B cells, T cells, PCs, and myeloid dendritic cells among PTLF, PTLR, and normal groups. (**E**).Representative H&E staining images showing the presence of TLS among PTLF, PTLR, and normal groups. Scale bars, 100 μm. (**F**).Dot plot showing the density of TLS of WSIs among PTLF, PTLR, and normal groups. (**G**).Volcano plots showing DEGs between PTLR and normal groups (GSE145780). (**H**).Bubble diagram depicting the signaling pathways enriched by KEGG analysis according to the upregulated DEGs in the PTLR group (GSE145780). (**I**).Volcano plots showing DEGs between PTLF and normal groups (GSE193135). (**J**).Bubble diagram depicting the signaling pathways enriched by KEGG analysis according to up-regulated DEGs in the PTLF group (GSE193135).

**
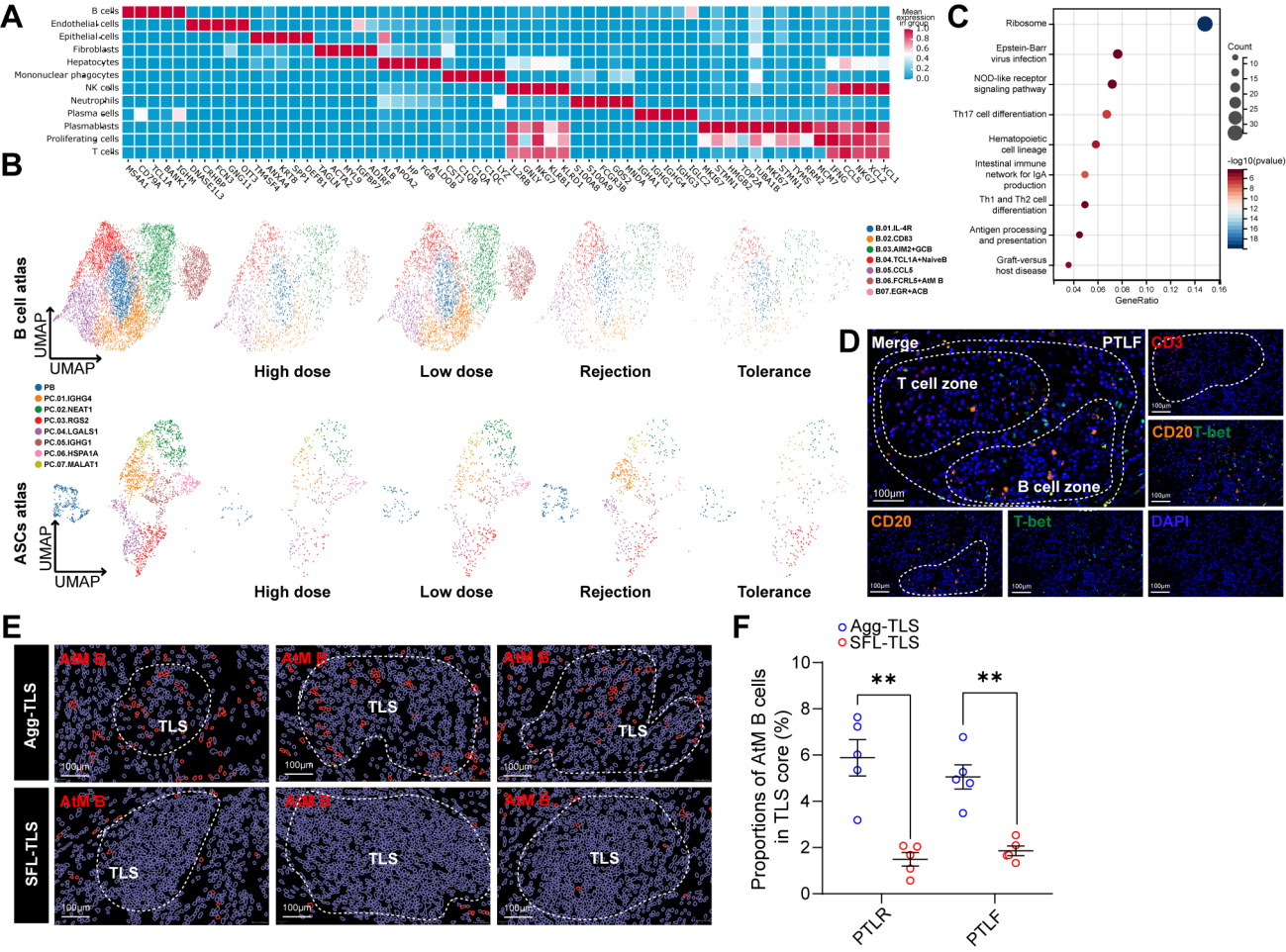
Figure S4. Single cell analysis unveils the AtM B cells promoting TLS formation in allograft rejection**

(**A**).Heatmap for expression of marker genes of identified 12 cell subsets. Color represents the maximum-normalized mean expression of cells expressing marker genes. (**B**).UMAP map depicting 7 clusters of B cells and 8 clusters of ASCs found in four groups after LDLT. (**C**).Bubble diagram depicting the signaling pathways enriched by KEGG analysis according to featured genes in IL-4R^+^ B cells. (**D**).Representative mIHC staining of differential abundance and spatial distribution features of AtM B cells in the TLS areas in PTLF livers. Scale bars, 100 μm. (**E**).Representative phenotype images of AtM B cells between Agg-, and SFL-TLS in PTLF livers. Scale bars, 100 μm. (**F**).Dot plot showing proportions of AtM B cells within TLS core between Agg-, and SFL-TLS in PTLR and PTLF livers.

**
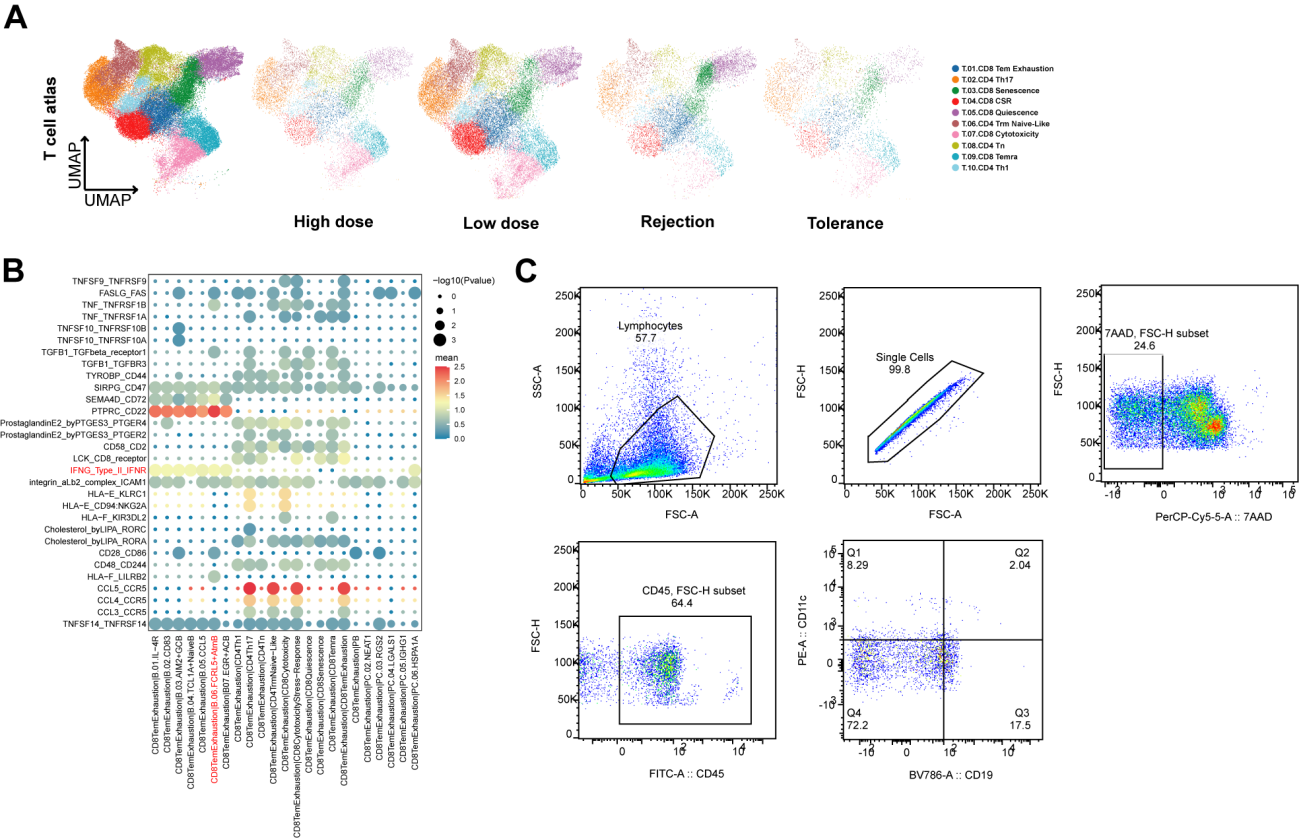
Figure S5. Exhausted CD8^+^ Tem cells promote AtM B cells differentiation via IFN-γ**

(**A**).UMAP map depicting 10 clusters of T cells found in four groups after LDLT.

(**B**).Bubble heatmap showing the mean interaction strength between the neighbor clusters at the boundaries for ligand-receptor pairs through Cellphonedb analysis. Dot size indicated the statistical significances by permutation test. Dot color indicated the mean interaction strength levels. (**C**).Representative flow cytometry plots of gating strategy for AtM B cells.

**
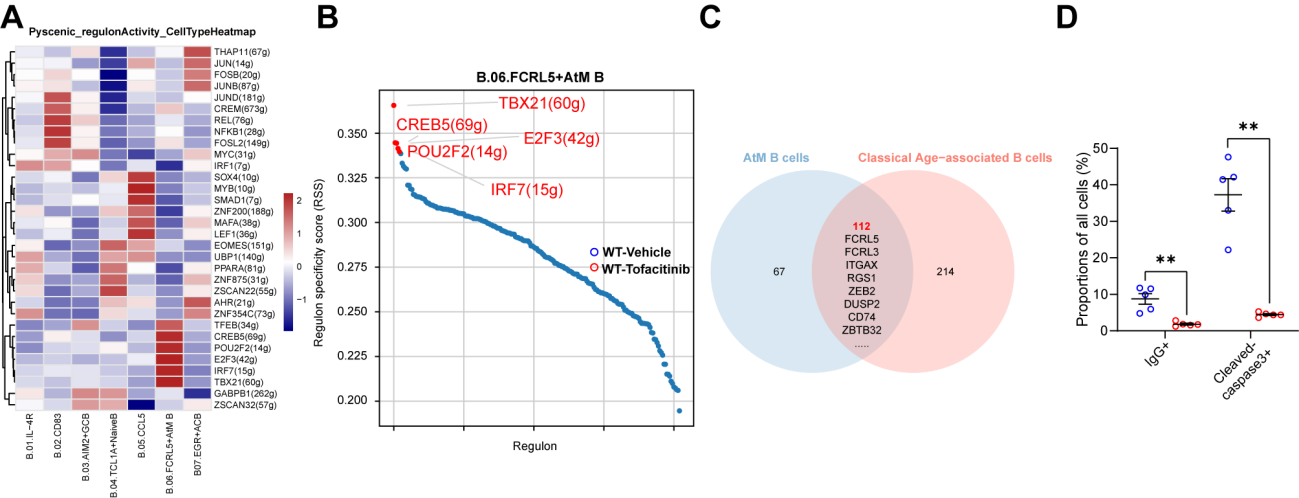
Figure S6. Target IFN-γ-JAK-STAT axis governs AtM B cell differentiation, TLS formation and alleviates allograft rejection**

(**A**).Heatmap showing key regulatory TFs identified by regulon specificity score (RSS) through pyscenic_regulon activity analysis. (**B**).Dot plot showing top 5 regulon specificity score across AtM B cells. (**C**).Venn diagram depicting the high concordance in featured genes was observed between ABCs and AtM B cells. (**D**).Dot plot showing the comparison of IgG^+^ cells and cleaved caspase-3^+^ apoptotic hepatocytes at day 14 in mice treated with and without Tofacitinib after MOLT (C57 (donor) to C3H (receptor)).
